# Isolation, Identification, and Selection of Bacteria With Proof-of-Concept for Bioaugmentation of Whitewater From Wood-Free Paper Mills

**DOI:** 10.3389/fmicb.2021.758702

**Published:** 2021-10-04

**Authors:** Nada Verdel, Tomaž Rijavec, Iaroslav Rybkin, Anja Erzin, Žiga Velišček, Albin Pintar, Aleš Lapanje

**Affiliations:** ^1^Department of Inorganic Chemistry and Technology, National Institute of Chemistry, Ljubljana, Slovenia; ^2^Department of Environmental Sciences, Jožef Stefan Institute, Ljubljana, Slovenia; ^3^Helmholtz-Zentrum Dresden-Rossendorf, Dresden, Germany; ^4^Faculty of Chemistry and Chemical Technology, Graduate School, University of Ljubljana, Ljubljana, Slovenia; ^5^Količevo Karton, d.o.o, Domžale, Slovenia

**Keywords:** *Aeromonas*, azo dye, bioaugmentation, principal component analysis, environmental microbiology, whitewater

## Abstract

In the wood-free paper industry, whitewater is usually a mixture of additives for paper production. We are currently lacking an efficient, cost-effective purification technology for their removal. In closed whitewater cycles the additives accumulate, causing adverse production problems, such as the formation of slime and pitch. The aim of our study was to find an effective bio-based strategy for whitewater treatment using a selection of indigenous bacterial isolates. We first obtained a large collection of bacterial isolates and then tested them individually by simple plate and spectrophotometric methods for their ability to degrade the papermaking additives, i.e., carbohydrates, resin acids, alkyl ketene dimers, polyvinyl alcohol, latex, and azo and fluorescent dyes. We examined correlation between carbon source use, genera, and inoculum source of isolates using two multivariate methods: principal component analysis and FreeViz projection. Of the 318 bacterial isolates, we selected a consortium of four strains (*Xanthomonadales bacterium* sp. CST37-CF, *Sphingomonas* sp. BLA14-CF, *Cellulosimicrobium* sp. AKD4-BF and *Aeromonas* sp. RES19-BTP) that degrade the entire spectrum of tested additives by means of dissolved organic carbon measurements. A proof-of-concept study on a pilot scale was then performed by immobilizing the artificial consortium of the four strains and inserting them into a 33-liter, tubular flow-through reactor with a retention time of < 15 h. The consortium caused an 88% reduction in the COD of the whitewater, even after 21 days.

## Introduction

The pulp and paper (P&P) industry is a major consumer and severe polluter of water resources. Particularly with wood-free paper, the production demands call for large volumes of water per ton of wood-free paper (Hamm and Schabel, 2007; Suhr et al., 2015). The trend in the P&P industry to reduce water consumption by completely closing the process water (whitewater) cycles (Hubbe et al., 2016) has not been adopted by the majority of paper mills (Suhr et al., 2015), since the resulting accumulation of additives used in papermaking adversely affects the production process.

To facilitate the closure of water cycles the whitewater can be treated to reduce the amount of accumulated organic matter and microbial growth (Hubbe et al., 2016). The form of the treatment is dependent on the type of paper production. In the wood-free paper industry, whitewater might be easier to treat, because it lacks recalcitrant woody compounds like lignin. On the other hand, several organic additives are used, which need to be effectively removed. Carbohydrates increase the biological oxygen demand, leading to the growth of mucus, while sizing and binding agents cause the formation of stickies, which impair the properties of the paper (Miranda et al., 2008; Licursi et al., 2016). Residues of azo dyes are unwanted visual contaminants and fluorescent stilbene dyes are recalcitrant and toxic to aquatic life (Salas et al., 2019). To the best of our knowledge, no specific treatment studies to address these issues associated with wood-free paper production have been conducted.

The best-known strategies to treat whitewater and reduce the amount of accumulating organic additives include membrane filtration, the use of coagulating chemicals, biological and enzymatic treatments, and the use of oxidizing agents (Hubbe, 2007; Suhr et al., 2015). These approaches have drawbacks, like membrane clogging, high sludge production, high energy consumption, low biodegradation of recalcitrant pollutants or the release of secondary pollutants (Priyadarshinee et al., 2016). Additionally, the main limitation of these in-mill, whitewater-treatment approaches is providing constant technological parameters, such as temperature and pH (Hubbe, 2007).

We focused on an alternative, biobased approach that could remove poorly degradable organic additives from wood-free whitewater that limit COD removal. Bioaugmentation with specific microorganisms can have minimal impact on the environment, reduce treatment costs, and can help remove even the most recalcitrant organic additives (Herrero and Stuckey, 2015; Santisi et al., 2015). State-of-the-art studies on the bioaugmentation of the P&P industry’s effluents have mainly focused on the degradation of recalcitrant lignin (Priyadarshinee et al., 2016), which is not present in wood-free whitewater. Those more relevant for the wood-free industry have demonstrated the microbial degradation of resin acids (Yu and Mohn, 2001, 2002) and a lowering of the total chemical oxygen demand using microbes (Malaviya and Rathore, 2007; Li et al., 2018; Majumdar et al., 2019). Additionally, ubiquitous microorganisms have been reported to hydrolyze starch (Mehta and Satyanarayana, 2016) and cellulose (Wierzbicka-Woś et al., 2019). More generally, microbes degrading azo dyes have been observed on several occasions (reviewed in Solís et al., 2012; Varjani et al., 2020) and the biodegradation of synthetic binders such as PVA has also been reported (Wu et al., 2019). Lastly, data on the bacterial biodegradation of AKD and fluorescent dyes is lacking, but their degradation might be feasible using the adapted indigenous inoculums from the whitewater environment (Atlas and Bartha, 1998; Malla et al., 2018).

If the start-up inoculums for biological treatment plants are sampled randomly from comparable environments (e.g., activated sludge from different treatment plants), they can perform poorly. Firstly, because according to Pareto’s law (Dejonghe et al., 2001; Cooper et al., 2019) only a minority of the sampled organisms will degrade the organic additives and, secondly, because the degraders of the recalcitrant additives will lose out to the faster-growing microorganisms that utilize the readily biodegradable additives. To prepare an optimal inoculum the selected bacteria need to be protected from competitors, cellular washing-off and fluctuating process conditions by the immobilization of cells onto porous carriers (Horemans et al., 2017a; Hassan et al., 2018).

Based on the available data, we hypothesize that bacterial degraders of the additives used in paper production are found in the whitewater itself. Most probably, the majority can decompose the most readily degradable carbon sources, such as starch, while some of them can degrade the less-biodegradable additives, even fluorescent dyes. Complex chemical mixtures require either the cooperation of several bacterial specialists that complement each other in different niches or a single generalist with versatile metabolic processes. It turns out that consortia are more efficient at degradation processes than single strains with broad repertoire of carbon sources (Santisi et al., 2015). It would, therefore, be ideal to acquire the smallest robust consortium consisting of highly active bacteria that occupy specific ecological niches defined by the nutrients they can utilize.

**FIGURE 1:**
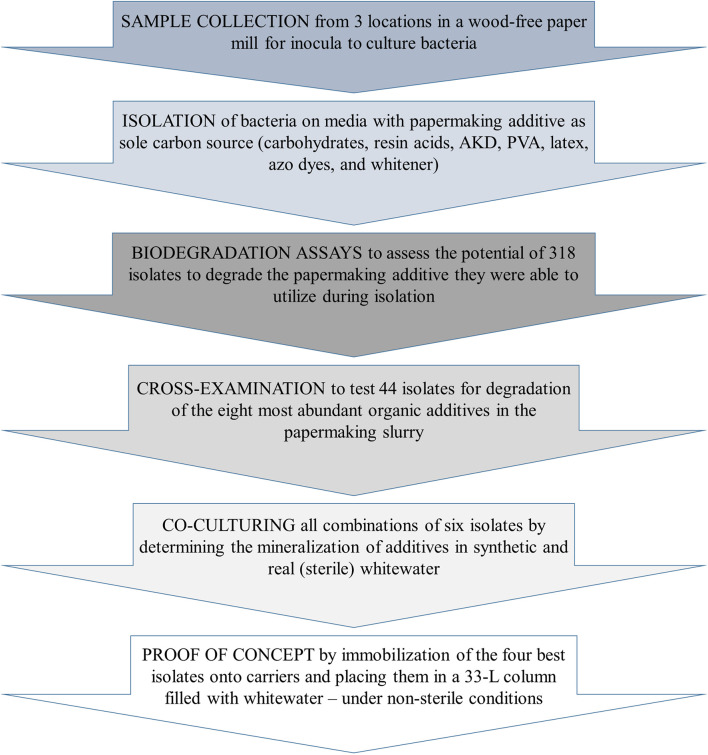
Construction of a bacterial consortium with high potential for wood-free whitewater treatment.

Accordingly, the main aim of our study was to simulate an actual whitewater microbial system and artificially construct a bacterial consortium capable of degrading all the additives that are introduced during the wood-free paper manufacturing process: starch, cellulose, resin acids, AKD, polyvinyl alcohol, latex, as well as azo and fluorescent dyes. To prepare such a consortium, we pursued several aims (Figure 1): (i) to collect bacteria with stable catabolism at low nutrient concentrations, we performed bacterial isolation under nutrient-poor conditions (Horemans et al., 2017b), (ii) to construct the optimal multi-strain inoculum, we selected bacteria according to their repertoire of carbon sources (i.e., the organic contaminants of whitewater) and prepared different combinations of active bacteria in synthetic and real whitewater and (iii) to upscale the prepared inoculum for a proof-of-concept pilot test, we applied a cell-immobilization strategy (Horemans et al., 2017a).

## Materials and Methods

### Whitewater Analysis

To better understand the stability of the characteristics of whitewater during the production process, we monitored several parameters of whitewater from a wood-free-paper mill over a period of 2 months (Supplementary Table A1). We measured the chemical oxygen demand (COD) (ISO 6060, 1989), the biological oxygen demand (BOD_5_) (ISO 5815-1, 2019) and the pH (pH meter model 827, Metrohm). The dissolved organic carbon content (DOC) was measured with an advanced TOC analyser (Teledyne Tekmar, model Torch) with a NDIR detector, by subtracting the measured inorganic carbon content from the measured total carbon content. The concentrations of anions (Cl^–^ and SO_4_^2–^) were measured using a Dionex DX-120 ion chromatograph (ISO 10304-1, 2007) and those of the cations (Ca^2+^, Mg^2+^, Na^+^, K^+^, and NH_4_^+^) using a Dionex Thermo Fisher Scientific ion chromatograph (ISO 14911, 1998). The content of HCO_3_^–^ anions was titrated with HCl (ISO 9963-2, 1994).

### Sample Collection

At the wood-free-paper mill we collected three types of samples: the clear filtrate (CF) of whitewater passed through the disc filter, the biofilm (BF) taken from the wet-end part of the paper machine and the effluent of the biological treatment plant (BTP). The CF and BF are both periodically washed into the biological treatment plant. We collected 1 liter of CF and BTP and 0.1 liter of BF and used them as inocula to culture the bacterial degraders of the papermaking organic additives. A total of 100 μL of the 10-times-diluted inoculum source was microbiologically enriched in M9 minimal medium (Miller, 1972) supplemented with a sole carbon source at final concentration 0.3 mM. M9 minimal medium contained 1x M9 salts (33.7 mM Na_2_HPO_4_ ⋅ 2H_2_O, 22.0 mM KH_2_PO_4_, 8.55 mM NaCl and 9.4 mM NH_4_Cl; VWR, United States), 1mM MgSO_4_ ⋅ 7H_2_O, 0.3 mM CaCl_2_ ⋅ 2H_2_O and 1x trace elements solution (all Sigma Aldrich, United States). We used 13 different carbon sources that are added to the paper-fiber slurry of the wood-free-paper production process and end up in the whitewater cycle (Hubbe et al., 2016): (i) the readily biodegradable additives (AKD, resin acids, cationic, native and soluble starch, cellulose, PVA, or latex), (ii) the non-readily biodegradable azo dyes (red, blue, black, and yellow), and (iii) the non-biodegradable fluorescent whitener (Supplementary Table A2). After 7 days at 25°C a total of 100 μL of the 100-fold-diluted culture enriched in the respective carbon source was plated in triplicates onto the solid M9 medium, supplemented with the respective carbon source. The obtained isolates were marked according to the carbon source they were able to utilize during isolation (Supplementary Table B1).

### Biodegradation Assays

For each bacterial isolate, the potential to degrade the papermaking additive used as a carbon source during enrichment and isolation was evaluated. The assay duration and the amount of carbon source in the medium were adjusted to determine the activity of the most potent degraders. The amount of each carbon source was adjusted so that discoloration of the test medium was visible, and the duration of the test was set to when the discoloration was visually apparent (Supplementary Tables A3, A4). Depending on the carbon source, biodegradation tests were performed in solid or liquid media (Supplementary Figure A1), all of which were supplemented with 10 mg/L cycloheximide (Sigma Aldrich, United States). Solid agar media were used to rapidly characterize the most active degraders of the readily biodegradable additives (see section “The Readily Biodegradable Additives”), while liquid media were used to characterize the degraders of the azo dyes and whitener (see section “Azo Dyes” and section “Fluorescent Whitener”). The absorption/emission maxima of the dyes and whitener were not affected by the concentration differences; therefore, the measurement was performed at a fixed wavelength (Supplementary Table A2). Centrifugation (12,000 × *g*, 5 min), which primarily served to sediment the cells, did not result in sedimentation in the assay for either the dyes or the whitener (see Supplementary Material section “Development of the Biodegradation Assays for Azo Dyes and Whitener”), but did in the case of the yellow dye, so the bioassays for this dye had to be adjusted accordingly (see section “Fluorescent Whitener”). Non-inoculated sterile media were used as controls in all experiments.

To test whether the degradation process of recalcitrant products is stable and suitable for industrial treatment process, we followed the time-dependent degradation of blue and red dyes and whitener using the most active bacterial strains. The exact initial concentrations of the carbon sources and the final duration of the tests are given in Supplementary Table A3.

#### The Readily Biodegradable Additives

The solid agar test media were based on the starch degradation assays described by Lal and Cheeptham (2012). A total of 0.25–10 g/L of starch, cellulose, AKD, resin acids, PVA, or latex was added to the M9 medium as the sole carbon source (Supplementary Table A3) and Lugol (I_2_+KI; Merck, Germany) was used to determine its degradation. The chemical reaction associated with this analysis is similar to the determination of iodine number. The formation of the iodine complexes by the addition of Lugol has been used to quantify the degradation of starch (Lal and Cheeptham, 2012), cellulose (Tashiro and Gakhutishvili, 2019), PVA (Procházková et al., 2014) and latex (Tyagi et al., 2000), whereas the degradation of AKD has not been previously studied using this approach. Therefore, for AKD degradation, a comparison was made with DOC measurements (see section “Characterization and Selection of Bacteria With the Highest Potential for Degradation of Organic Additives”). For each biodegradation assay, a single colony was inoculated on a circular area (*r* = 0.5 cm) on the solid M9 medium in 2-4 replicates (Supplementary Figure A1E), and degradation was performed for up to 15 days at 25°C (Supplementary Table A4). Lugol was then poured onto the agar medium, and after 10 min the radius of the clearance zone around the colonies (*r*_*DIS*_) was measured and the relative degradation coefficient calculated by normalizing the degradation to a fixed maximum value for all media using the equation *c*/*c*_*0*_ = (*r*_DIS_^*2^−*r*_*DIS*_^2^)/*r*_DIS_^*2^. *r*_DIS_^*^ for the biodegradation assays was 3 cm, while for cross examination tests (see section “Characterization and Selection of Bacteria With the Highest Potential for Degradation of Organic Additives”) it was the discoloration radius of the best degrader strain per carbon source.

As the growth of several cultures was difficult to detect visually due to the opacity of the M9 test plates, the viability of each strain was checked by re-growing in nutrient broth (NB) to avoid false negatives.

#### Azo Dyes

The discoloration experiments with red, blue, and black azo dyes were performed as cometabolism experiments in NB medium supplemented with 6–57 mg/L of the respective dye (Supplementary Table A3). By cometabolism we mean the simultaneous biodegradation of the organic substances making up the medium NB and the dye additive. NB was added to the dyes medium, because in M9 media with 47–95 mg/L of dye as sole source of carbon no clear results were obtained: no visual discoloring appeared, only minor decrease in intensity for inoculated media, decrease in intensity of sterile media for 5% and no increase in OD_600_ in 10–13 days. The discoloration experiments were performed according to Parmar and Shukla (2018), with modifications, in transparent untreated 96 flat-bottom microplates (VWR), where 200 μL of each test medium was inoculated with a single bacterial strain. Bacteria were incubated until the appearance of the first visual discoloration, 18–68 h, at 50 rpm and 25°C, the cells that discolored the dye were pelleted by centrifugation (12,000 × *g*, 5 min) and the supernatant was used for spectrophotometric evaluation of the discoloration of the liquid. The dye concentration was determined at the specific absorption maximum of a particular dye (Supplementary Table A2) using a Synergy H4 spectrophotometer (Biotec, United States) and the prepared calibration curves (Supplementary Figures A1, A2). The coefficient *c*/*c*_0_ was calculated, where *c* represents the unused and *c*_0_ represents the initial concentration of the carbon source.

For the yellow dye biodegradation assay, M9 medium supplemented by the yellow dye (95 mg/L) was used to measure the total absorption by the dye and cells (*c*), while M9 containing a small amount of glucose (47 mg/L) was used to measure only the contribution of cells (*c*_*cells*_). The concentration of the remaining dye was calculated using the equation (*c*-*c*_*cells*_)/*c*_0_, where *c*_0_ denotes the initial concentration of yellow dye. Measurements were performed at 400 nm after incubation at 25°C and 50 rpm for 7 days. In addition, the OD_600_ was recorded for each sample.

#### Fluorescent Whitener

Cometabolism tests with the whitener were performed in M9 supplemented with 4 g/L glucose (M9_*Glc*_) and 0.9 g/L whitener, to determine the most potent degraders. The duration of the assay was set at the time when distinct visual differences in the opacity were observed, and a fourfold increase in OD_600_ was measured simultaneously for the strongest strain. Bacterial cells were removed from media with distinct visual differences in opacity by centrifugation (12,000 × *g*, 5 min), and the 50-fold diluted supernatant was transferred to black flat-bottomed 96-well microtiter plates (VWR). Whitener concentration was determined by fluorescence measurements using Synergy H4 set at 315 ± 6 nm (excitation) and 340-550 nm (emission), with emission maximum at 430 nm, endpoint read type, normal read speed, probe vertical offset 8 mm, and 100% sensitivity.

### Identification

Using 16S rRNA gene sequencing, we identified all the isolates that were shown to degrade the corresponding organic additive above the detection limit of the biodegradation assays, described in section “Biodegradation Assays” (Supplementary Table B2). A further 50, 52, 17, and 75% of isolates enriched and isolated on media containing red, blue, black or whitener dye, respectively, were identified as being able to use the recalcitrant dyes as sole carbon sources regardless of the detection limit of the biodegradation assays (Supplementary Table B1N). Overall, the DNA of 95 bacterial strains was obtained using isolation with Chelex^®^100 Resin (Walsh et al., 1991). A single colony of each isolate cultured on solid NB was transferred into the chelex solution. After a 15-min incubation at 95°C and a 15-min centrifugation at 4°C and 5000 rpm the supernatant containing the DNA was transferred into a sterile microcentrifuge tube and stored at −20°C. The 16S rRNA gene was PCR amplified using standard primers according to Hubad and Lapanje (2013). The primers 27f (AGA GTT TGA TCM TGG CTC AG) and 1492r (CGG TTA CCT TGT TAC GAC TT) (Integrated DNA Technologies, United States) were used for amplification. The PCR products were sequenced using Sanger technology with the forward primer 27f at Macrogen, NL.

### Phylogenetic Trees

The chromatograms of the 16S rRNA sequences were curated in Chromas 2.6.6 (Technelysium Pty. Ltd.) to produce 1090–1340 bp large sequences. The sequence identity was obtained using BLASTN 2.9.0+ (Camacho et al., 2009) and the databases RDP_11 (Ribosomal Database Project) and NCBI (National Center for Biotechnology Information). The sequences were deposited in the NCBI GenBank database with the accession numbers MW144826-MW144947 and MW131116.

Two types of phylogenetic trees were created on the basis of the Kimura 2-parametric model in MEGA X software (Kumar et al., 2018). The clustering of 95 isolates together with the most similar type strains was inferred using the unweighted pair-group method with arithmetic mean (UPGMA). The evolutionary distances were computed using the p-distance method, whereas the clustering of the four isolates of the consortium together with the most similar type strains was conducted using the NJ algorithm.

### Principal Component Analysis and FreeViz Projection

Statistical correlation between carbon source use, genera, and inoculum source of isolates was examined using two multivariate methods: principal component analysis (PCA) and FreeViz projection (Demšar et al., 2007) using Orange version 3.27.1 software (Demšar et al., 2013). We used the relative reciprocal values of *c*/*c*_0_ (see Supplementary Table B3) on a scale from 0 to 1: *c*_*U*_/*c*_0_, where *c*_*U*_ indicates the relative amount of carbon source utilized after a given time and higher values indicate greater degradation activity. We defined the extreme degraders as those strains that degraded fluorescent whitener up to *c*_*U*_/*c*_0_ > 70% and other additives up to *c*_*U*_/*c*_0_ > 50%. Supplementary Table B4A shows the 11 variables (degradation activities) and their 44 average values. We calculated incomplete data for the tests in solid media using a model-based imputer of the software Orange, and for the cometabolism test of blue in NB we set a value of zero (see Supplementary Table B4C). Since we were dealing with many variables, we visualized the main features obtained by linear projection of PCA and FreeViz by explicitly making the basis vectors visible only when their endpoints exceeded a certain distance from the center, and marking the area where the basis vectors are not displayed with a circle, as previously reported (Demšar et al., 2007; Sakurai et al., 2020).

### Characterization and Selection of Bacteria With the Highest Potential for Degradation of Organic Additives

Based on the results of the biodegradation assays (see section “Biodegradation Assays”), we selected 44 of the most active strains, 2–5 per carbon source (Supplementary Table A4). These were then cross examined for their ability to degrade eight organic papermaking additives (cationic and soluble starch, cellulose, resin acids, AKD, red and blue azo dyes, and whitener) (Supplementary Figure A2), which are most abundant in the paper-fiber slurry of a wood-free paper mill (Supplementary Table A2). In this way, we were able to characterize metabolic specialists, who degrade only a single additive, and metabolic generalists, who degrade a broad repertoire of organic papermaking additives. Using principal component analysis (see section “Principal Component Analysis and FreeViz Projection”), we selected six isolates covering the entire repertoire of additives: (i) starch, (ii) the readily biodegradable, (iii) dyes in glucose, (iv) dye in NB, (v) dye in NB, cellulose and whitener, and (vi) all additives. Activity for whitener was a preferred characteristic as it is the most difficult to degrade. For each of the six strains, the CFU versus OD_600_ parameters were calibrated by culturing the strains in NB for 48 h at 25°C (Supplementary Figure A3).

The cross-examination was performed using biodegradation assays (see section “Biodegradation Assays”), with experimental conditions as similar as possible for all additives. To prepare the inoculum, isolates were cultured for 3 days at 25°C and 50 rpm in M9_*Glc*_ medium. Tests with the non-readily biodegradable compounds were performed for 4 days at 25°C as cometabolism tests in M9_*Glc*_ supplemented with 5 mg/L red or blue dye or the whitener or in NB media supplemented with 5 mg/L blue dye (see Supplementary Table A3); the cometabolism of the blue dye was tested in NB and M9_*Glc*_ because the discoloration of dyes varies in different media (Solís et al., 2012).

The tests with the readily biodegradable compounds were performed in liquid and solid M9 media. The tests in the liquid M9 media supplemented with 0.5 g/L cationic or soluble starch or 4 g/L AKD lasted 4 days at 25°C. The absorbance was recorded at 584 nm before (*c*_*cells*_) and after (*c* and *c*_0_) the addition of Lugol and the coefficients (*c*-*c*_*cells*_)/*c*_0_ were compared (see section “Biodegradation Assays”). Tests with solid M9, to which either 5 g/L cationic starch or cellulose or 0.25 g/L AKD or resin was added, lasted 7 days at 25°C with a fixed volume of medium per Petri plate (see Supplementary Table A3).

Overall, to select bacteria with the highest potential for bioaugmentation of wood-free whitewater, we used detection methods based on absorption and fluorescence spectroscopy, and measuring the discoloration radius after addition of Lugol, whereas for media containing suspended solids (whitewater) (see section “*In vitro* Co-culturing Test” and section “Pilot-Scale Experiment Proof of Concept Only”) we determined the mineralization of organic additives by DOC and COD measurements (Table 1).

**TABLE 1:** Selection tests for building an artificial consortium and analytical methods to evaluate degradation of the additives.

Test	Additive^a^	Analytical method for assessing the degradation of the additive
Biodegradation assay (see section “Biodegradation Assays”)	CST, ST, NST, CE, RES, AKD, PVA, and LX	Addition of Lugol, *r*_*DIS*_ measurement
	RED, BLU, BLA, and Y	Absorbance at specific dye maximum
	WH	Fluorescence at specific dye maximum
Cross-examination (see section “Characterization and Selection of Bacteria With the Highest Potential for Degradation of Organic Additives”)	CST, ST, and AKD	Addition of Lugol, absorbance at 584 nm
	RED and BLU	Absorbance at specific dye maximum
	WH	Fluorescence at specific dye maximum
	CST, CE, AKD, and RES	Addition of Lugol, *r*_DIS_ measurement
*In vitro* co-culturing under sterile conditions (see section “*In vitro* Co-culturing Test”)	synthetic whitewater (CST, PVA, RES, and azo dye direct blue 15)	DOC measurement
	industrial whitewater (all additives)	
Proof of concept under unsterile conditions (see section “Pilot-Scale Experiment Proof of Concept Only”)	industrial whitewater (all additives)	COD measurement

*^*a*^Abbreviations of additives are listed in Supplementary Table A2.*

### *In vitro* Co-culturing Test

All 63 combinations of the six selected strains (single strains and co-cultures of two, three, four, five and six strains) were used to test the degradation of organic additives in synthetic and in real industrial whitewater under sterile conditions. Co-culturing in synthetic whitewater was performed to increase the regularity of the test, since the composition of the real whitewater depends on the papermaking process. The co-culturing test helped us to select the best combination for mineralization of additives in real whitewater. For the preparation of non-complex synthetic whitewater, M9 medium was used, to which CST, PVA, resin, and Direct blue 15 dye were added in a weight ratio of 1:1:1:0.5, totaling 107 mg/L DOC. Real whitewater was first sterilely filtered through a 0.2-μm membrane polypropylene filter (Macherey-Nagel) and then, M9 salts were added to achieve a 1× final concentration. Cells of each strain were collected by centrifugation (12,000 × *g*, 5 min) from cultures grown overnight in NB. Then they were washed three times and resuspended in sterile 0.9% NaCl. The number of cells in the suspension was calculated for each strain based on the calibration curves (see section “Characterization and Selection of Bacteria With the Highest Potential for Degradation of Organic Additives”). The volume of each strain suspension was adjusted to achieve a concentration of 10^7^ CFU/mL in the final 200-μL co-culture test mixture. The co-culture experiment was performed in six replicates in 96-well microtiter plates. After 3 days at 25°C, the media were sterilely filtered through 0.2-μm polypropylene filters. Complete mineralization of organic carbon was analyzed by DOC measurements using a TOC analyzer (Shimadzu) (see Supplementary Table B5).

In addition, for the final four selected strains used to construct the artificial consortium, AKD degradation was confirmed by absorbance and DOC measurements (Supplementary Table A5).

### Pilot-Scale Experiment (Proof of Concept Only)

The pilot-scale experiment was performed under unsterile conditions, for the proof of concept only. To demonstrate feasibility, the artificial consortium of the four bacteria selected in the co-culturing tests was first immobilized on porous carriers, which were then placed in a 33-liter plexiglass column (*r* = 0.06 m, *h* = 2 m) (Supplementary Figures A4A–C). A parallel, continuous flow of industrial whitewater was introduced into the column at a rate of 2 L/h using a Qdos 30D peristaltic pump (Watson Marlow). The hydraulic retention time in the column was estimated to be 13–15 h. Aeration was set up at the bottom of the column and controlled by a Tecsis compression force transducer (Wika Group). Oxygen saturation of the whitewater was monitored using a HQ40d model multi portable oxygen electrode (Hach) submerged 20–30 cm below the top of the column. During the run, there was minimal movement of the carriers caused by mixing of the liquid with the aeration system. After 8 days, a recirculation was set up at the same flow rate with an additional peristaltic pump to direct the flow back into the column. After 10 days, a new batch of whitewater was introduced. To create favorable growth conditions for the bacteria a ratio of COD : Total N : Total *P* = 100: 5: 1 was achieved by adding urea (solid) and H_3_PO_4_ (85% w/v) (both Sigma Aldrich, United States) to the whitewater. The following process parameters were measured at the influent and effluent using Nanocolor rapid tests (Mesherey-Nagel): Total N, Total P, PO_4_^3–^, NO_3_^–^, NH_4_^+^, and COD. The pH was measured using a portable pH meter model HQ11d (Hach). Samples of 0.25-L were collected at the inflow and outflow of the column in clean plastic containers and the process parameters were measured immediately after sampling. The control system was as described by Qu et al. (2005). It was performed in 250 mL flasks containing 190 mL of clean carriers, a proportional amount of carriers immersed into 0.5% sodium alginate (2 carriers per 250 mL), and 250 mL of unsterile industrial whitewater in triplicate. The flasks were incubated in a shaker incubator at 50 rpm and 25°C, and the COD was measured before and after incubation of 15 h, i.e., the duration of the retention time, and the results were used as a control (Supplementary Table B6A).

Bacterial cells were immobilized on the carriers as previously reported (Horemans et al., 2017a). The four isolates were grown separately for 72 h at 25°C in liquid NB and the cells were collected by centrifugation (15 min at 12,000 × *g*) and re-suspended in 9 g/L NaCl to yield 3 × 10^8^ CFU/mL of BLA14 and 1–4 × 10^9^ CFU/mL of CST37, AKD4, and RES19. Sodium alginate at a final concentration of 0.5% (w/v) and 0.4 L of sterile Kaldnes K3 carriers (Tongxiang Small Boss Ltd., China) were added to the bacterial suspension, which was then vacuumed three times using a two-stage P4Z rotary pump (ILMVAC) to impregnate the inner surface of the porous carriers with the cell suspension. We achieved 3–4 × 10^6^ CFU/carrier for BLA14, CST37 and RES19 and 2 × 10^7^ CFU/carrier for AKD4. A total of 1.6 L of bacteria- loaded carriers, 0.4 L per isolate, was mixed with 23.4 L of clean carriers (25 L in total) and stored in a container for 16 h before being packed into the column. It was expected that cells released from the impregnated carriers would colonize the clean carriers in the system according to Horemans et al. (2017a). We therefore assessed the CFU/carrier^∗^ for each isolate, as if the cells would ideally be distributed from the immobilized to the total volume of carriers in the column (further details in Supplementary Table B6B). The relative difference in CFU/carrier after 7 days was calculated for each isolate individually (Supplementary Table B6B). For this purpose, the CFU/carrier of the freshly immobilized and the 7-day-old carriers collected 20–30 cm below the top of the column were determined using standard plate counting. A total of four carriers were placed in 50 mL sterile centrifuge tubes containing 20 mL sterile 9 g/L NaCl, and all experiments were performed in two replicates. Cells were removed from the carriers by vortexing the carriers at maximum speed for 5 min. The amount of each was determined using a stereomicroscope (Motic SMZ-168) (Supplementary Figure A4E).

Monitoring the removal efficiencies of individual organic additives was performed indirectly due to the complexity of whitewater composition, containing low amount of additives, and suspended solids and dyes that interact with light absorption, by inferring the degradation of each additive from the characterized activities of the four isolates (see section “Extreme Degraders Originate From Every Inoculation Source” and section “By selecting Six Isolates We Can Degrade the Whole Repertoire of the Organic Additives”).

## Results

### Results of Systematic Analysis of the Whitewater

Systematic analysis of the whitewater from a wood-free paper mill over a 2-month period revealed relatively constant COD, BOD_5_ and DOC, of 300, 165, and 95 mg/L, respectively (±10%, *n* = 19, 8, 19) (Supplementary Table A1). Consequently, we prepared a whitewater treatment solution using an artificial consortium of preselected indigenous bacterial strains that efficiently degrade organic additives used in papermaking.

### The Phylogeny of Bacterial Isolates Relates to the Inoculation Source

**FIGURE 2:**
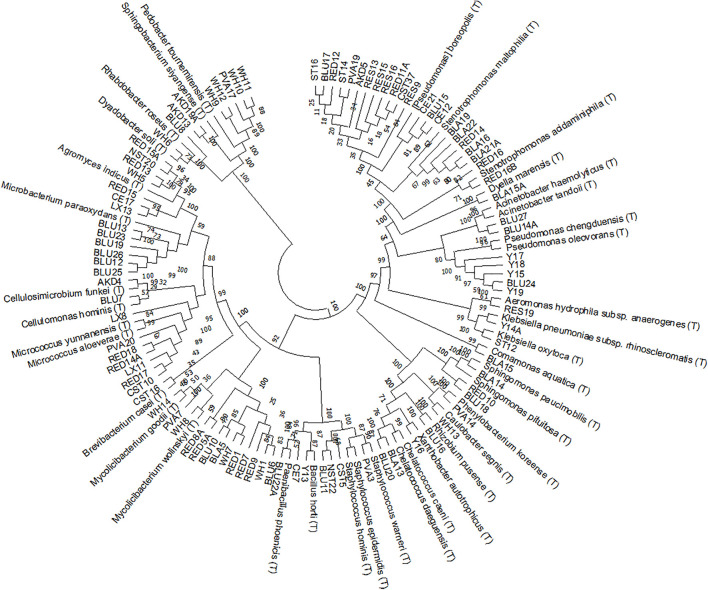
Phylogenetic tree UPGMA (unweighted pair-group method with arithmetic mean) based on 16S rRNA sequences of 95 isolates with their closest type strains. Values at the bifurcations show the bootstrap values of 500 clustering replications.

We isolated a total of 318 bacterial strains from three inoculation sources, from which we successfully identified 95 by 16S rRNA gene sequencing. The isolates belonged to four phyla: Proteobacteria, Firmicutes, Actinobacteria and Bacteroidetes (Figure 2). The largest number of them, 15 isolates, was identified with the closest relative [*Pseudomonas*] *boreopolis* (T), currently classified as *Xanthomonadales bacterium*, followed by 13 isolates as *Mycobacterium* sp. and 7 isolates as *Micrococcus* sp., *Stenotrophomonas* sp. and *Agromyces* sp. (Table 2). The highest proportion of all the identified isolates was obtained from CF (55%) and, respectively, 25% and 20% from BF and BTP. We found that isolates belonging to the same genus were in the majority (86%) inoculated from the same inoculation source (Table 2), except for *Micrococcus* sp. and *Staphylococcus* sp. that we assess as primary contaminants from the paper mill.

**TABLE 2:** The distribution of genera of the sequenced isolates among the inoculation sources [clear filtrate (CF), biofilm (BF), and effluent from the biological treatment plant (BTP)]; only genera with more than one isolate are listed.

Genus / taxonomic group^a^	Number of identified isolates (percentage of identified isolates)		
	CF	BF	BTP
*Sphingomonas*	3 (100%)	0 (0%)	0 (0%)
*Pedobacter*	5 (100%)	0 (0%)	0 (0%)
*Chelatococcus*	2 (100%)	0 (0%)	0 (0%)
*Xanthomonadales bacterium*	14 (93%)	1 (7%)	0 (0%)
*Mycobacterium*	12 (92%)	0 (0%)	1 (8%)
*Micrococcus*	5 (71%)	2 (29%)	0 (0%)
*Stenotrophomonas*	0 (0%)	6 (86%)	1 (14%)
*Agromyces*	0 (0%)	6 (86%)	1 (14%)
*Pseudomonas*	0 (0%)	5 (100%)	0 (0%)
*Microbacterium*	0 (0%)	0 (0%)	6 (100%)
*Acinetobacter*	0 (0%)	0 (0%)	2 (100%)
*Sphingobacterium*	0 (0%)	0 (0%)	2 (100%)
*Klebsiella*	1 (50%)	0 (0%)	1 (50%)
*Paenibacillus*	1 (50%)	0 (0%)	1 (50%)
*Staphylococcus*	2 (50%)	1 (25%)	1 (25%)
**Total**	**45 (55%)**	**21 (25%)**	**16 (20%)**

*^*a*^Isolates were identified by 16S rRNA gene sequencing (see section “Identification”).*

### Highly Active Degraders of the Organic Additives Are Found Among the Isolated Bacteria

Preliminary characterization of 318 bacterial isolates for their ability to degrade the carbon source they used during isolation (Supplementary Table B1) helped us to select 44 strains that were best able to degrade individual carbon sources (Supplementary Table A4). Overall, 30 of the selected strains degraded one of the readily biodegradable additives (cellulose, starch, latex, PVA, and AKD) and 15 of them degraded one of the five dyes (red, blue, black, yellow, and whitener). *Xanthomonadales bacterium* spp. were found to be the most active strains for degradation of readily biodegradable additives CST, ST, AKD, PVA, and CE with 5–72% *c*/*c*_0_ higher degradation compared to other strains. For resin acids, latex and native starch, *Aeromonas* sp. RES19-BTP, *Agromyces* sp. strain LX13-BF and *Agromyces* sp. NST20-BTP were the most active with 36%, 44% and 4% more *c*/*c*_0_ than others, respectively.

**FIGURE 3:**
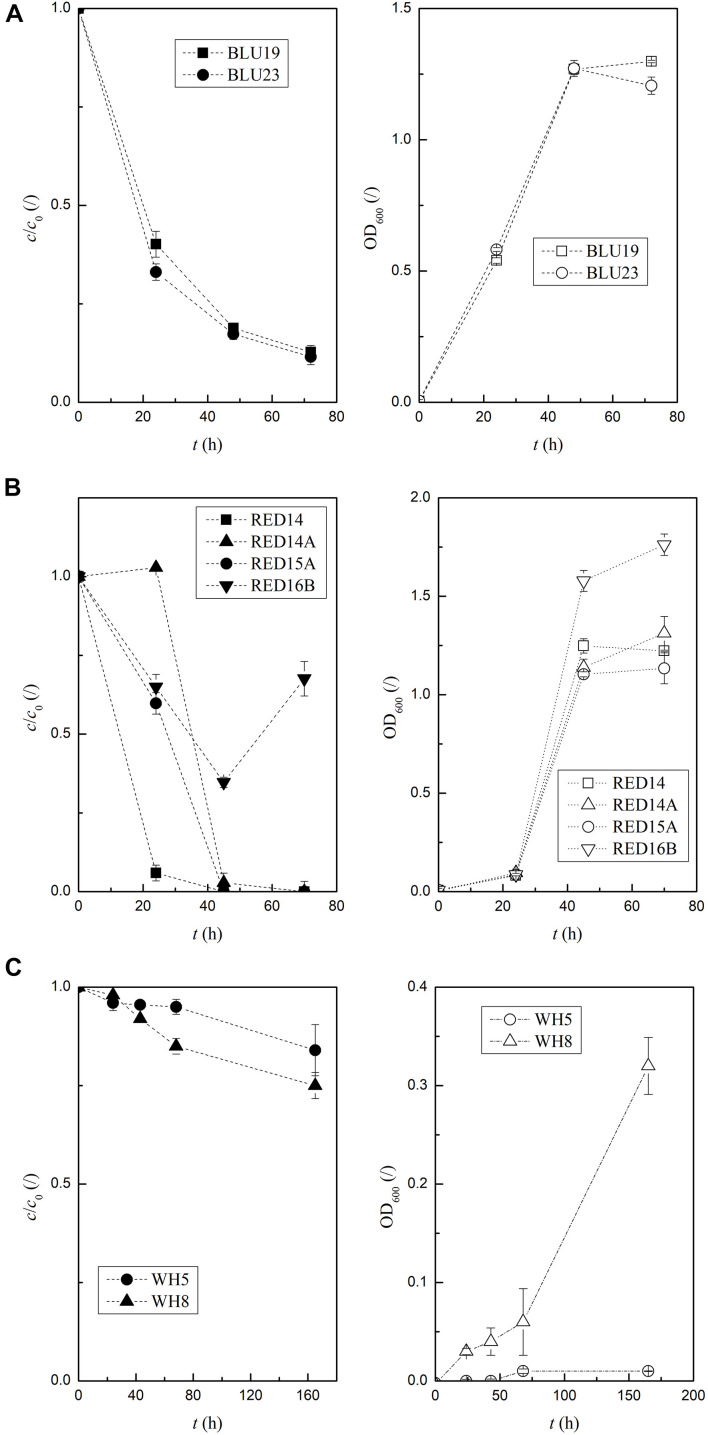
Time-dependent degradation activity of the most active isolates for red, blue, and whitener incubated up to 165 h at 25°C. The error bars indicate the standard errors of triplicates; low values of *c*/*c*_0_ indicate a high degradation activity; *c*/*c*_0_ are full and OD_600_ empty symbols. **(A)** BLU19 and BLU23 in NB+10 mg/L blue. **(B)** RED14, RED16B, RED14A, and RED15A in NB+13 mg/L red. **(C)** WH5 and WH8 in M9_*Glc*_+0.5 g/L whitener.

According to the results of the biodegradation assay, the two isolates selected for blue dye degradation, BLU19 and BLU23, degraded 80% and 60% *c*/*c*_0_, respectively, after 18 h (Supplementary Table A4). Determination of time-dependent activities (using different preparation of inoculum, see Supplementary Table A3) revealed that BLU19 and BLU23 still showed significant differences after 24 h, degrading 40 ± 3% and 33 ± 2% *c*/*c*_0_, respectively (Figure 3A and Supplementary Table A6). However, after 48 h, a linear decrease to 13% ± 0% *c/c_0_* was achieved, and we observed no differences in degradation between the two BLU isolates. At 24 h, RED14 showed 9–16 times faster red dye discoloration and degraded 96% of the dye (Figure 3B), while at 48 h there were no significant differences between the RED isolates (14, 14A, 15A) that degraded all of the dye. Since we used a ∼100-fold higher concentration of whitener, this resulted in higher *c*/*c*_0_ values (Figure 3C). Therefore, we could not directly compare the *c*/*c*_0_ values between the degraders of whitener and the red and blue dyes. OD_600_ measurements of WH5 and WH8 revealed no or minimal growth in 165 h, which is in contrast with CFU counts obtained, as 10^9^ and 6 × 10^10^ CFU/mL of WH5 and WH8 were counted, respectively. This indicates a disruption of light absorption by whitener. In the first 72 h of incubation, the maximum degradation of whitener to 75% *c*/*c*_0_ was recorded for WH8, which was 12% more than the others.

We found that the most active yellow isolates, Y17, Y18, Y19, degraded yellow dye similarly in 7 days, up to 55 ± 5% *c*/*c*_0_, and Y14A up to 92% *c*/*c*_0_ (Supplementary Table A4). In 72 h the black dye was discolored up to 75 ± 6% *c*/*c*_0_ by three isolates with an extremely high OD_600_ of 1.8–2.4.

### Extreme Degraders Originate From Every Inoculation Source

**FIGURE 4:**
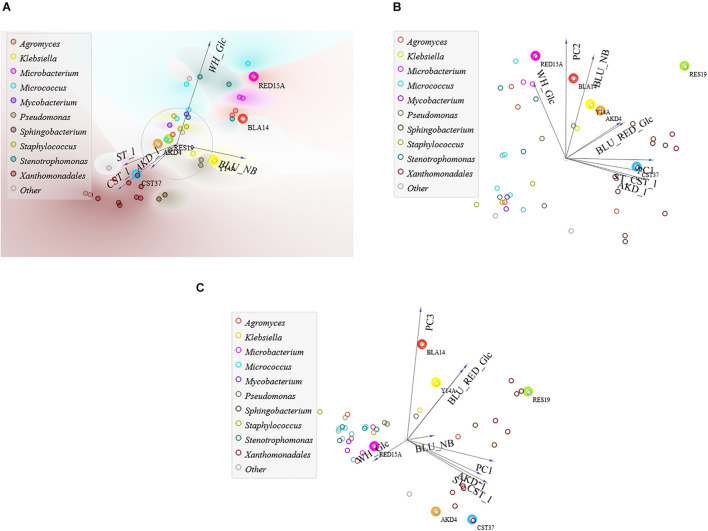
FreeViz and PCA of tests for the repertoire of carbon sources of 44 isolates in liquid media with genera for clusters. **(A)** FreeViz projection and **(B)** PC1PC2 with 77% and **(C)** PC1PC3 with 62% explained variances. RED15A (circled in pink), BLA14 (red), Y14A (yellow), RES19 (green), AKD4 (brown), and CST37 (blue).

Using multivariate PCA and FreeViz projection, we found that papermaking additives could be classified into three groups: whitener, the least biodegradable additive, azo dyes and readily biodegradable additives (Figure 4A and Supplementary Figure A5A). Of the 44 isolates tested for carbon source repertoire, 70, 45, and 29% from the inoculation sources BTP, CF, and BF, respectively, are extreme degraders of at least one type of additive (Supplementary Table B4B). Starch utilization is a distinctive feature of isolates from CF, with 50 % (out of 20 isolates) being extreme degraders of readily biodegradable additives. Isolates from BF are characterized by the utilization of whitener, with 29% (out of 14) being extreme degraders of whitener. In the case of BTP, we isolated extreme degraders of all additives.

With 82% explained variances (Supplementary Table B4E), a positive correlation was found between the test results in the solid and liquid media with CST and AKD, with 86 ± 3% equal amounts of above- and below-average degraders (Supplementary Table B4F). Among them, seven isolates are extreme degraders of CST, AKD, resin and cellulose in solid media, while RES19 and RED15A are also extreme degraders of whitener. About 70% of extreme cellulose degraders are also extreme degraders of AKD, resin and CST, while 69% of extreme starch degraders are also extreme degraders of AKD when tested in liquid media. In addition, a positive correlation was found between dye degraders in M9_*Glc*_, with 82% of above and below average blue and red dye degraders occurring in equal proportions, with only three isolates causing extreme degradation of both dyes: BLA14, RES19, and AKD5. *Xanthomonadales bacterium* spp. stand out as extreme degraders of all readily biodegradable additives (Supplementary Figures A5C,D). RES19 is the best generalist, with an average *c*/*c*_0_ of 30% ± 7% for all additives.

### By Selecting Six Isolates We Can Degrade the Whole Repertoire of the Organic Additives

**FIGURE 5:**
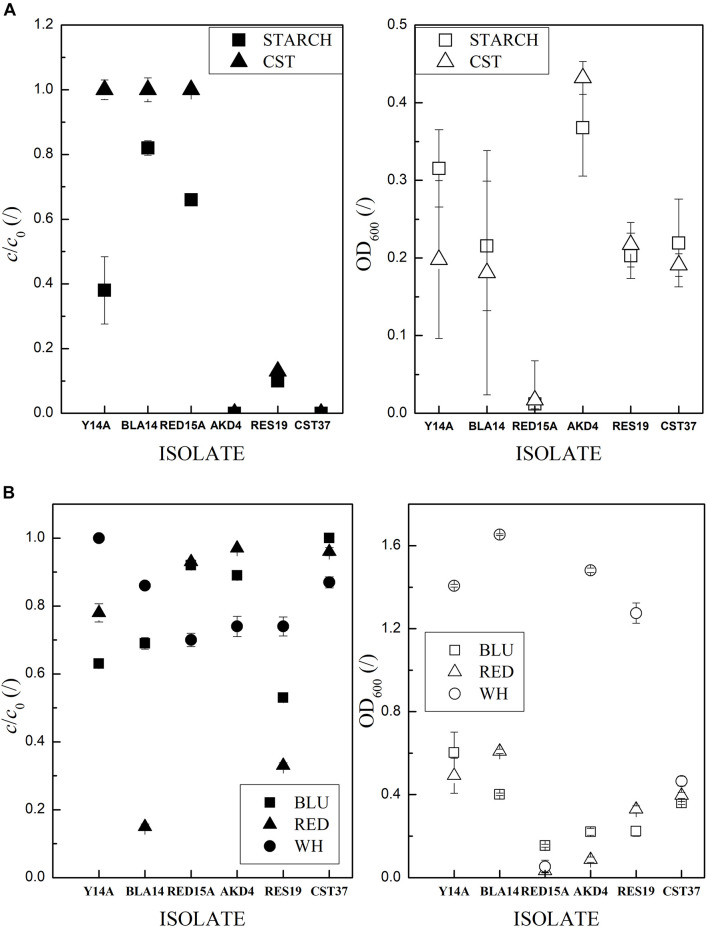
Reservoir of carbon sources of the six selected isolates. Error bars indicate the RSE of triplicates. Low values of *c*/*c*_0_ indicate high degradation activity. OD_600_ are empty and *c*/*c*_0_ full symbols. **(A)** Starch and **(B)** dyes and whitener in M9_*Glc*_.

Strain AKD4 was selected on the basis of high hydrolysis of starch in liquid media (Figure 5A) and whitener (Figure 5B), with 0% and 74% *c*/*c*_0_ after 3 days, respectively, with the closest type strains *Cellulosimicrobium funkei* (T) and *C. cellulans* (T) (Supplementary Figure A6C). *Xanthomonadales bacterium* strain CST37-CF, which is closest to the [*Pseudomonas*] *boreopolis* (T) type strain (Supplementary Figure A6A), also hydrolyzed 100% starch. It was selected because it was among the most active for all readily biodegradable additives, with an average *c*/*c*_0_ for starch, cellulose, resin acids and AKD of 18% ± 11% and, unlike other comparable strains, active for utilizing whitener with 87% *c*/*c*_0_ (Supplementary Table B3). BLA14 and RES19 had the highest average activity for azo dyes (37% ± 22% *c*/*c*_0_ and 39% ± 8%, respectively). BLA14 was selected as a specialist for dye utilization, while RES19 was selected as the most active general isolate, an extreme degrader of starch (in liquid) (1% ± 1% *c*/*c*_0_) and an extreme degrader of whitener, red and blue dye with an average 48% ± 10% *c*/*c*_0_. BLA14 has the closest type strain *Sphingomonas paucimobilis* (T) (Supplementary Figure A6B) and RES19 is related to *Aeromonas dhakensis* (T) and *A. caviae* (T) (Supplementary Figure A6E). Strains Y14A, AKD13, WH5 and RED15A were the most active (12% ± 0.3% *c*/*c*_0_) for the blue dye in NB (Supplementary Table B3D). Among them, we selected Y14A with related strains *Klebsiella pneumoniae* (T) and *K. quasipneumoniae* (T) (Supplementary Figure A6D) because it was the most active for the blue dye in NB (11% *c*/*c*_0_) and could also utilize the yellow dye as the only carbon source. RED15A with its sister strain *Agromyces indicus* (T) (Supplementary Figure A6C) was selected because, unlike AKD13, it is an extreme degrader of whitener (70% *c*/*c*_0_) and a general degrader. It degraded starch, resin acids, AKD and cellulose in agar media with an average *c*/*c*_0_ of 36% ± 4% (Supplementary Table B3G).

### Coexistence of the Selected Strains Is Possible *in vitro*

Co-culturing in media with synthetic and industrial whitewater was compared for all 63 combinations of the six selected isolates under sterile conditions (Supplementary Table B5). It was found that the isolate combinations consumed more organic carbon than the individual isolates after 3 days. Similar isolate combinations were most active in both media, showing that we correctly assumed the composition of the synthetic whitewater. We selected the most active combination in industrial whitewater media, with a *c*/*c*_0_ of 62.5 ± 1.5% in both media, which consisted of a consortium of CST37, BLA14, RES19, and AKD4 (Supplementary Table B5). The regularity of the co-culture test was confirmed as the same combinations of strains were the best degraders in both media. The low degradation rates were attributed to the low amount of M9 salts in the industrial medium and the large amounts of the dye in the synthetic whitewater. The best consortium degraded 29% ± 3% more carbon content than the single CST37 and was 23% more active in industrial whitewater than the single extreme general degrader RES19.

### The Immobilized Artificial Consortium of Four Isolates Performs Well in Proof-of-Concept Pilot Test

The pilot experiment was conducted under non-sterile conditions using industrial whitewater and carriers loaded with strains of the artificial consortium, CST37, BLA14, AKD4, and RES19, placed in a 33-liter column (Supplementary Figures A4A–C). After the flow stabilized, COD decreased from an initial 183 mg/L in the influent to 59 mg/L in the effluent, corresponding to a *c*/*c*_0_ of 32%. No decrease in COD was recorded for the control system after 15 h (see section “Pilot-Scale Experiment Proof of Concept Only”). Initially, a large coefficient of 2.7 was calculated for COD between the unfiltered and filtered effluent. As expected, the difference became insignificant after 3 days, and even on the 21st day, the COD of a new batch of whitewater decreased from 400 to 50 mg/L, corresponding to a *c*/*c*_0_ of 13%. Thus, we achieved 88% COD removal (Supplementary Figure A4D and Supplementary Table B6A). Initially, 1.6 L of carriers, including 0.4 L for each isolate, were immobilized with 3–4 × 10^6^ CFU/carrier for RES19, CST37, and BLA14 and 2 × 10^7^ CFU/carrier for AKD4, which extrapolates to 4–7 × 10^4^ and 3 × 10^5^ CFU/carrier, respectively, for 25 L carriers (Supplementary Table B6B). On the seventh day, 10% of unknown microorganisms were detected on the used carriers taken 20 cm below the top of the column, a 22% ± 1% increase in CFU/carrier for RES19 and AKD4, 3% for dye specialist BLA14 and 56% for CST37. From the characterization results in Figure 5 and Supplementary Table A4 we extrapolate that the readily biodegradable additives were mineralized to 10% *c*/*c*_0_, whereas mineralization of dyes was almost inexistent, 98% *c*/*c*_0_ of the COD, with discoloring of red and blue dye to 60% *c*/*c*_0_ and minimal discoloring of the recalcitrant whitener, black and yellow dye.

## Discussion

We have demonstrated that the artificially constructed consortium of four indigenous bacterial strains, i.e., *Xanthomonadales bacterium* strain CST37-CF, *Sphingomonas* sp. BLA14-CF, *Cellulosimicrobium* sp. AKD4-BF and *Aeromonas* sp. RES19-BTP, is capable of degrading the organic additives used in a wood-free production process (Supplementary Figure A4D). As we surmised from Horemans et al. (2017a), the strains of the artificial consortium that were immobilized on the carriers also spread on the surfaces of the newly added clean carriers (section “The Immobilized Artificial Consortium of Four Isolates Performs Well in Proof-of-Concept Pilot Test”) and the stable degradation activity of the artificial consortium was confirmed by a 13% *c*/*c*_0_ for the COD (88% COD reduction), even on day 21 of the experiment, with no decrease in the COD for the control experiment. Our results in 15 h retention time are promising compared to the COD reduction obtained in other bioaugmentation studies relevant for wood-free paper mill whitewater treatment (Yu and Mohn, 2001, 2002; Malaviya and Rathore, 2007; Li et al., 2018; Majumdar et al., 2019).

Microorganisms derived from the same environment, e.g., activated sludge sample, would be a poorer starting inoculum for the whitewater treatment because according to the biodegradation assay results (Supplementary Table B1) the 318 isolates somewhat conform to the Pareto principle (Dejonghe et al., 2001; Cooper et al., 2019), i.e., 82% of the isolates are below-average degraders of the papermaking additives. Already in the laboratory tests, the four selected bacteria were among the best in degrading the separate organic additives used in papermaking (Figure 5). Two of the isolates are extreme degraders of different types of additives (CST37 and BLA14), and two are generalists (RES19 and AKD4). The co-culturing tests that yielded the artificial consortium of four bacteria (Supplementary Table B5) confirmed the results of Santisi et al. (2015), i.e., that more bacteria have an advantage over a single extreme generalist such as strain RES19. As expected, BLA14, specialist in azo dyes, lose out to the faster growing degraders of the readily biodegradable compounds (Supplementary Table B6B). Accordingly, with extrapolating results from Figure 5 and Supplementary Table A4 we assume that 90% of the readily biodegradable additives were mineralized and 40% of red and blue dye discolored, while the recalcitrant whitener, black and yellow dye remained in the whitewater.

As we suspected according to Malla et al. (2018), microbes from a wood-free paper mill are suitable for use of the pollutants at hand. In clear filtrate, isolates were continuously supplied with papermaking additives, while in biofilm, isolates were exposed to nutrient deprived conditions and had to adapt to utilization of recalcitrant whitener (Supplementary Figure A5B). Accordingly, the PCA and FreeViz results show that the *Xanthomonadales bacterium* strains from CF stand out as highly active degraders of readily biodegradable additives (Figure 4 and Supplementary Figures A5C,D). On the other hand, *Micrococcus* sp. RED14A- BF (from BF) is specialized for the recalcitrant whitener (Supplementary Table B4A), in agreement with Cui et al. (2012). Additionally, in agreement with Solís et al. (2012), the discoloration of non-readily biodegradable dyes by the isolates is dependent on the media (Figure 4), as discoloration in glucose is more dye-specific, whereas in NB dye-degrading enzymes could be stochastically excreted. Accordingly, RED16B, in agreement with Assih et al. (2002), produced stained products in NB (Figure 3B) that could interfere with the whitewater treatment process.

The results for the degradation of readily biodegradable additives by *Xanthomonadales bacterium* sp. strain CST37-CF were confirmed by the reported (Guo et al., 2018) high activity for PVA utilization and discoloration of dyes by its closest relative, [*Pseudomonas*] *boreopolis*. However, there are no previous reports on its utilization of AKD, resin acids and cellulose. The discoloration of dyes by *Klebsiella pneumoniae* (Zablocka-Godlewska et al., 2015; Abdulsalam et al., 2020) and the utilization of polycyclic aromatic hydrocarbons by *Sphingomonas paucimobilis* (Lu et al., 2019; Ravintheran et al., 2019) are consistent with our results for *Klebsiella* sp. strain Y14A-BTP and *Sphingomonas* sp. strain BLA14-CF. Lignocellulolytic enzymes secreted by *Cellulosimicrobium cellulans* (Liu et al., 2015; Dou et al., 2019), starch hydrolysis and degradation of phthalates by *Agromyces* sp. (Li et al., 2003; Zhao et al., 2016) and the broad metabolic capabilities of *Aeromonas hydrophila* (Seshadri et al., 2006; Ogugbue and Sawidis, 2011; Thomas et al., 2020) are in agreement with our results for *Cellulosimicrobium* sp. strain AKD4-BF, *Agromyces* sp. strain RED15A-BF and *Aeromonas* sp. strain RES19-BTP. Thus, review of the literature on the closest relatives of the six isolates selected by PCA confirmed the accuracy of our selection methods.

In short, the strategy for the preparation of an artificial consortium for bioaugmentation and the proposed improvements are as follows:

1. Collect an environmental sample and isolate 300–400 bacteria. Isolating the bacteria with stable catabolism at low nutrient concentrations (Horemans et al., 2017b) was performed at nutrient levels, comparable to a 500-fold diluted lysogeny broth. This was most time-consuming step because growth of the isolates was slow, which is in agreement with Gray et al. (2019).

2. Test the activity of the isolates with respect to the degradation of the targeted organic additives. Since simple, unambiguous selection methods for complex media are lacking, we propose to develop new analytical methods using specialist bacteria.

3. Find the combinations that can best be implemented in the desired environment.

4. Test the best combination in the desired environment for proof of concept. The pilot-scale experiment can only be treated as a proof of concept (see section “Pilot-Scale Experiment Proof of Concept Only”), nevertheless, we assume that application of the artificial consortium for whitewater treatment would reduce the amount of stickies and improve the paper production efficiency (Miranda et al., 2008; Licursi et al., 2016).

5. Test the stability of the consortium over time using different immobilization procedures and carriers, which according to the literature (Horemans et al., 2017a; Hassan et al., 2018) significantly affect the washout of the microbes.

6. Upgrade for industrial application by testing the bioreactor scalability (Connelly et al., 2017).

## Conclusion

• The four bacteria selected for bioaugmentation were immobilized on carriers and placed in a 33-L column filled with whitewater. Even on day 21, an 88% decrease in COD was measured with a retention time of approximately 15 h. The results show that these bacteria can purify the whitewater of a wood-free paper mill.
• The four selected bacteria are two specialists, i.e., *Xanthomonadales bacterium* sp. CST37-CF and *Sphingomonas* sp. BLA14-CF, which complement each other in different niches, and two general degraders, i.e., *Cellulosimicrobium* sp. AKD4-BF and *Aeromonas* sp. RES19-BTP.
• 318 isolated bacteria adapted to the wood-free paper production process were mostly below-average degraders of the organic additives tested. They were isolated from three interrelated inoculation sources: whitewater, biofilm from the paper machine and effluent from the biological treatment plant. In most cases, the inoculation sources determined the genera of the isolated bacteria.
• We made the following new findings: (a) In most cases, starch degraders are also active in the degradation of alkyl ketene dimers, (b) the discoloration of dyes in different substrates is uncorrelated, and (c) the *Xanthomonadales bacterium* sp. is characterized by high activity for readily biodegradable additives.

## Data Availability Statement

The sequences were deposited in the NCBI GenBank database with the accession numbers MW144826–MW144947 and MW131116.

## Author Contributions

NV, AL, AP, and TR contributed to conception and design of the study. NV and TR organized the database and performed the statistical analysis. NV, IR, AE, and ŽV performed experimental work. NV wrote the first draft of the manuscript. NV, AL, and TR contributed to manuscript revision. All authors approved the submitted version.

## Conflict of Interest

The authors declare that the research was conducted in the absence of any commercial or financial relationships that could be construed as a potential conflict of interest.

## Publisher’s Note

All claims expressed in this article are solely those of the authors and do not necessarily represent those of their affiliated organizations, or those of the publisher, the editors and the reviewers. Any product that may be evaluated in this article, or claim that may be made by its manufacturer, is not guaranteed or endorsed by the publisher.

## Abbreviations

16S rRNA: 16S ribosomal ribonucleic acid
BOD_5_: biological oxygen demand after 5 days
CFU: colony-forming units
COD: chemical oxygen demand
DOC: dissolved organic carbon
OD_600_: absorption at 600 nm
PCA: principal component analysis
PVA: polyvinyl alcohol
(T): type strain
AKD: alkyl ketene dimer
BF: biofilm
BLA: black dye HM 2482
BLU: blue dye
BTP: effluent from the biological treatment plant
*c*/*c*_0_: coefficient between concentration of unutilized vs. initial carbon source
*c*_*u*_/*c*_0_: relative reciprocal values of *c*/*c*_0_ on a scale from 0 to 1
CE: cellulose
CF: clear filtrate
CST: cationic starch
DB15: direct blue 15 dye
M9_*Glc*_: M9 minimal medium supplemented with 4g/L glucose
LX: latex
NB: nutrient broth No. 1
NST: native starch
RED: direct red 253 dye
RES: resin acids
ST: starch
WH: whitener
Y: yellow dye.
