# Supplementary material for: Isolation, Identification, and Selection of Bacteria With Proof-of-Concept for Bioaugmentation of Whitewater From Wood-Free Paper Mills
Source: Front Microbiol. 2021 Oct 4;12:758702. doi: 10.3389/fmicb.2021.758702 (PMC8521037; doi:10.3389/fmicb.2021.758702)
Supplement: Supplementary file 1 [file Data_Sheet_1.pdf]

Supplementary Material A

A.1 Development of the biodegradation assays for azo dyes and whitener

First, the influence of centrifugation on sedimentation of dyes was tested by determining the absorption or emission intensity of the dyes before and after centrifugation (12,000×g, 5 min). For whitener, excitation at 315 nm showed a maximum wavelength emission at 440 nm, as for 4,4'-diamino-2,2'-stilbenedisulfonic acid (DSDS), and the coefficient between the fluorescence intensity after and before centrifugation (I_{cent}/I) was 98%. For black dye an absorption decrease of 4% at both peak maxima was recorded, for red, blue and direct blue 15 dye (DB15) no decrease in absorption was detected and yellow dye showed a decrease of 50%. Calibration curves for all tests in liquid media were generated for molar concentrations at λ_{max} and the volume of liquid in microplates was kept constant to ensure accurate absorbance measurements. To quantitatively determine the amount of unhydrolyzed dye by measuring the absorption of supernatants, the tests with NB in microplates had to be completed in a maximum of three days. To determine the activities of the most active bacteria, initially, relative standard error between the triplicates was maximum 3%. During incubation the microtiter plates were covered with sterile lids and were packed in bags to prevent evaporation. Wet paper was inserted into the bags to maintain a constant humidity.

References

Hočevar, B., Grilc, M., and Likozar, B. (2019). Aqueous Dehydration, Hydrogenation, and Hydrodeoxygenation Reactions of Bio-Based Mucic Acid over Ni, NiMo, Pt, Rh, and Ru on Neutral or Acidic Catalyst Supports. *Catalysts*, *9*, 286-306. https://doi:10.3390/catal9030286

Marshall, C. J., and Kunkle, C. A. (1992). Mineral dye pigments. U.S. Patent No 5,106, 421.

Supplementary Table A.1 Whitewater analysis of the wood-free paper mill within a period of two months with average, minimum and maximum values.

**t (day)[a]**	**COD (mg/L)**	**DOC (mg/L)**	**BOD_5 (mg/L)**	**HCO_3^- (mg/L)**	**Cl^- (mg/L)**	**NO_3^- (mg/L)**	**SO_4^{2-} (mg/L)**	**Na^+ (mg/L)**	**K^+ (mg/L)**	**NH_4^+ (mg/L)**	**Ca^{2+} (mg/L)**	**Mg^{2+} (mg/L)**	**pH**
**AVG**	**303**	**94**	**166**	**153**	**40**	**8**	**179**	**78**	**0**	**2**	**59**	**15**	**7.5**
**MIN**	**190**	**31**	**80**	**50**	**25**	**1.6**	**109**	**62**	**0**	**1.7**	**41**	**10**	**6.8**
**MAX**	**500**	**175**	**254**	**269**	**62**	**15**	**309**	**95**	**1.5**	**3.5**	**88**	**29**	**8.2**
**-1**	232	134	158	165	49	7.8	183	78	0	2.5	52	16	7
**1**	214	54		130	34	15	175	62	0		54	16	7.1
**2**	278	52	80	114	25	13	309	67	0		88	17	7.5
**5**	190	36		111	26	7.4	267	90	0	2	78	13	7.2
**6**	210	31		90	26	8.4	274	86	0	1.8	79	16	7.5
**7**	200	37		50	26	9.1	233	70	0		59	14	6.8
**8**	268	77	109	141	36	9.1	233	95	0	2.2	67	14	7.5
**9**	320	91		156	45	3.7	132	66	0		43	17	7.3
**12**	252	78		123	39	5.7	117	66	0	2.5	45	12	7.6
**13**	356	95		144	36	7.7	156	78	0		52	13	7.6
**14**	300	120	178	184	46	8.2	125	85	0	2.7	51	13	7.4
**15**	284	105		180	41	4	138	76	0	1.7	64	14	7.8
**16**	345	114		170	44	6	109	75	0	2	41	10	7.7
**21**	362	128		141	36	6.2	150	76	0	2	60	13	7.5
**22**	500	175	180	168	44	2.3	116	76	0	2.1	50	11	7.3
**24**	243	74		167	27	4.3	122	76	0		60	12	7.4
**28**	448	128	183	146	57	13	235	84	0	1.7	61	16	8.2
**31**	393	120	254	253	52	11.1	171	82	0	2.5	52	14	7.4
**59**	371	145	183	269	62	1.6	163	87	1.5		71	29	8.2

[a] … -1 refers to one day before cleaning of the whitewater system and days 1-59 to sampling after restarting of the system.

Supplementary Table A.2. Organic additives used as carbon sources with abbreviation, their assumed shares in the industrial wood-free paper fiber slurry, calculated according to production sheets of four different paper grades of a wood-free paper mill and chemical structure with molar mass to calculate the calibration curves; for dyes maximum wavelength absorption or excitation/emission and dye intensity before and after centrifugation at 12,000×g.

Abbr.	Share (%)	Chemical structure
CE	4	Microcrystalline cellulose (VWR, USA) CAS 9004-34-6; M ($C_{12}H_{22}O_{11}$)=342 g/mol (monomeric unit)
CST	0.4	Cationic potato starch, degree of derivatization 0.04, based on integrals in ^{1}H-NMR; M($C_6H_{10}O_5$)=162 g/mol (monomeric unit)
RES	0.4	Rosin, CAS No 8050-09-7; contains abietic and pimeric type resin acids; M($C_{20}H_{30}O_2$)=303 g/mol
AKD	0.18	Alkyl ketene dimers: 2-oxetanone, 3-C12-16-alkyl-4-C13-17-alkylidene derivatives, CAS No 84989-41-3; M=477 g/mol
WH	0.10	Whitening agent; fluorescent brightening agent; tetrasodium 2,2'-ethene-1,2-diylbis[5-({4-[bis(2-hydroxyethyl)amino] -6-[(4-sulfonatophenyl)amino]-1,3,5-triazin-2-yl}amino]benzenesulfonate]; spectral properties similar to 4,4'-diamino-2,2'-stilbenedisulfonic acid; M($C_{42}H_{44}N_{12}Na_4O_{16}S_4$)=1193 g/mol; excitation at 315 nm with maximum emission at 430 nm; I_{cent}/I = 98%
NST	0.005	Native soluble starch, CAS 9005-25-8; (2R,SS,4S,5R,6R)-2-(hydroxy methyl)-6-[(2R(xy-oxane-3,4,5-triol,3S,4R,5R,6S)-4,5,6-tri hydroxy-2-(hydroxymethyl)oxan-3-y]oxy-oxane-3,4,5-triol; M($C_6H_{10}O_5$)=162 g/mol (monomeric unit)
ST	/	Starch, soluble (Sigma Aldrich, USA) CAS 9005-84-9; M($C_6H_{10}O_5$)=162 g/mol (monomeric unit)
RED	0.005	Direct red dye 253 (Marshall 1992), CAS 142985-51-1: Dinatrium;(3*E*)-7-[[4,6-bis(2-hydroxyethylamino)-1,3,5-triazin-2-yl]amino]-4-oxo-3-[[4-[(4-sulfonatophenyl)diazenyl]phenyl] hydrazinylidene] naphthalene-2-sulfonate; M($C_{29}H_{26}N_{10}Na_2O_9S_2$)=769 g/mol; λ_{max}=510 nm; I_{cent}/I = 100%
BLU	0.005	Blue dye, similar biodegradation to direct blue 15, CAS No. 2429-74-5. To evaluate the structural similarity of the dye BLU to DB15, cometabolism tests on 44 selected strains were compared for the biodegradation of BLU and DB15 dye in NB. After 72 h similar discoloration patterns were found. Direct blue 15 is 2,3,3'-[(3,3'-dimethoxy-4,4'-

		biphenylylene)bis(azo)]bis [5-amino-4-hydroxy-tetrasodium salt]; $M_{DB15}(C_{34}H_{28}N_6NaO_{16}S_4)$=927 g/mol; $\lambda_{max\ BLU}$=560 nm; $\lambda_{max\ DB15}$=600 nm; I_{cent}/I = 100%
BLA	0.003	Black dye HM 2482, CAS No. 89857-06-7; 1,3'-bipyridinium,5'-[[4-[[7-[(1',2'-dihydro-6'-hydroxy-3,4-dimethyl-2'-oxo[1,3'-bipyridinium]-5'-yl)azo]-1-hydroxy-3-sulpho-2-naphthalenyl]azo]phenyl]azo]-1'-[3-(dimethylamino)propyl]-1',2'-oxo-, salt with 2-hydroxypropanoic acid; $M(C_{50}H_{57}N_{11}O_{14}S_4)$=1068 g/mol; λ_{max}=440, 590 nm; I_{cent}/I = 96%
Y	0.003	Yellow dye containing C; H; N; O; S; stilbene derivative; M^{a}=180 g/mol; λ_{max}=400 nm; I_{cent}/I = 50%
PVA	<0.001	Polyvinyl alcohol, CAS 9002-89-5; $M(C_2H_4O)$=44 g/mol (monomeric unit)
LX	<0.001	Latex dispersion, styrene-butadiene copolymer; $M(C_{12}H_{14})$=158 g/mol (monomeric unit)

[a] Unknown structure, molar weight taken for glucose.

Supplementary Table A.3. Protocols with inoculum preparation and composition of media.

Test	Inoculum preparation	Carbon source	Selection media
Biodegradation assays (*see* Section 2.3)	Colony of the isolate on NB agar was picked and transferred to a circle on selection media. Incubation was at 25 °C for 7-13 days.	starch	M9 10 g/L ST
			M9 10 g/L CST
			M9 10 g/L NST
		CE	M9 5 g/L CE
		RES	M9 0.25 g/L RES
		AKD	M9 0.25 g/L AKD
		PVA	M9 5 g/L PVA
		LX	M9 0.25 g/L LX
	Colony of the isolate on NB agar was picked and transferred into M9 media with 47 mg/L of appropriate dye or whitener. After incubation at 25 °C for 3 days 10 µL of the culture was transferred into the selection medium.	BLU	NB + 6 mg/L BLU
		RED	NB + 15 mg/L RED
		BLA	NB + 57 mg/L BLA
		WH	$M9_{Glc}$ + 0.9 g/L WH; supernatant 20-times diluted

		Y	M9 95 mg/L yellow dye and M9 47 mg/L glucose
Time-dependent degradation determination	A colony was transferred into corresponding M9 media with 47 mg/L of appropriate dye or whitener. After incubation at 25 °C for 3 days 10 µL of the culture was transferred into the selection medium.	RED	NB + 13 mg/L RED
		BLU	NB + 10 mg/L BLU
		WH	$M9_{Glc}$ + 0.5 g/L WH; supernatant 20-times diluted
Tests for the repertoire of carbon source usage (*see* Section 2.7)	A colony was transferred into $M9_{Glc}$ for 3 days at 25 °C. 10 µL of the culture was transferred into the selection medium.	CST	M9 0.5 g/L CST
		ST	M9 0.5 g/L ST
		AKD	M9 4 g/L AKD
		RED	$M9_{Glc}$ + 5 mg/L RED
		BLU	$M9_{Glc}$ + 5 mg/L BLU
		WH	$M9_{Glc}$ + 5 mg/L WH
		BLU	NB + 5 mg/L BLU
		CE	20 mL / plate; M9 agar with 5 g/L CE
		CST	15 mL / plate, M9 agar with 5 g/L CST
		AKD	20 mL / plate, M9 agar with 0.25 g/L AKD
		RES	20 mL / plate, M9 agar with 0.25 g/L RES
In vitro co-culturing test (*see* Section 2.8)	The growth of the six selected isolates was calibrated with determining cfu vs. OD_{600}. Overnight culture was harvested in NB, cleared three times in 9 g/L NaCl. OD_{600} was measured and a calibration curve was generated. The amount of volume was adjusted so that an equal initial sum of bacteria per each medium was added, i.e. 10^7 CFU/mL.	synthetic whitewater	M9 (CST, PVA, RES, DB15); in the ratio CST : PVA : RES : DB15 = 1 : 1 : 1 : 0.5
		industrial whitewater	M9 sterile whitewater

Proof of concept pilot test (*see* Section 2.9)	Separate overnight cultures of the four bacteria of the consortium harvested in NB, cleared in 9 g/L NaCl, OD_{600} measured and CFU calculated.	industrial whitewater	whitewater (unsterile) with added urea and H_3PO_4 in the ratio COD : N : P = 100 : 5 : 1

Supplementary Table A.4. Selected isolates, active for the carbon source which they used during isolation with numbers of isolated bacteria per carbon source and results of biodegradation assays - C/C_0 and OD_{600} values and test durations.

**Carbon source**	**Number of isolates**	**Active isolate**	**C/C_0 (%)**	**OD_{600} (/) ± RSE(%)**	**t (d)**	**C_0 (g/L)**
Cationic starch	44	CST37	75	/	9	10
		CST5	83	/		
		CST10	83	/		
		CST16	83	/		
Native starch	31	NST20	83	/	11	10
		NST22	87	/		
Starch, soluble	35	ST12	72	/	13	10
		ST14	0	/		
		ST16	0	/		
Resin	20	RES19	39	/	7	0.25
		RES9	75	/		
		RES13	75	/		
		RES15	75	/		
AKD	27	AKD4	51	/	7	0.25
		AKD5	46	/		
		AKD13	75	/		
		AKD19A	75	/		
PVA	20	PVA19	13	/	7	5
		PVA20	75	/		
		PVA3	75	/		
		PVA7	75	/		
		PVA14	83	/		
Cellulose	22	CE12	0	/	11	5
		CE21	0	/		
		CE7	81	/		
		CE17	46	/		
Latex	23	LX13	31	/	7	0.25
		LX11	75	/		
		LX8	84	/		
Red	20	RED14	0	1.4±4	2	0.015
		RED14A	20	1.5±5		
		RED15A	0	1.0±9		
		RED16B	11	1.7±1		
Blue	27	BLU19	80	0.6±7	0.75	0.006
		BLU23	60	1.6±5		
Whitener	12	WH8	59	0.13±2	3	0.9

		WH5	99	0.04±7		
Yellow	14	Y19	50	0.02±0	7	0.095
		Y17	53	0.02±1		
		Y18	60	0.01±1		
		Y14A	92	0.01±1		
Black	23	BLA14	70	1.8±2	2.8	0.057
		BLA16	70	2.4±3		
		BLA19	84	2.3±3		

Supplementary Table A.5. AKD degradation confirmed by decrease in absorption and DOC removal, presented in C/C_0 values; the bacteria were inoculated for 62 h, initially 10^8 cfu/mL and C_0 4 g/L.

**Isolate**	**C/C_0 (%) ± RSE (%)**	
	**Absorption**	**DOC**
CST37	56±0	62±0
AKD4	57±0	66±1
RES19	56±0	71±1
BLA14	100±1	100±1

Supplementary Table A.6. Results of the time-dependent activity of the most active red, blue and whitener dye degraders for the dye they used during isolation at 25 °C. Degradation is presented in percentages of C/C_0 values ± relative standard errors of triplicates. WH5 and WH8 after 165 h were 10^9 and 6×10^{10} CFU/mL, respectively.

**Dye**	**Isolate**	**$C/C_{0\ \lambda max}$ (%)±RSE (%)**				**OD_{600} (/)±RSE (%)**				
		**24 h**	**48 h**	**72 h**	**165 h**	**0 h**	**24 h**	**48 h**	**72 h**	**165 h**
BLU	**BLU19**	40±3	19±1	13±2	/	0.01±0	0.6±1	0.9±2	0.9±1	/
	**BLU23**	33±2	17±1	12±2	/	0.01±0	0.6±1	0.9±3	0.8±3	/
RED	**RED14**	6±3	-7±2	-15±1	/	0.01±1	0.1±1	1.3±4	1±0.5	/
	**RED14A**	103±0	2.8±3	-14±3	/	0.02±2	0.1±6	1.1±2	1±8.5	/
	**RED15A**	60±3	-16±0	-16±1	/	0.01±1	0.6±6	1.1±1	1±8	/
	**RED16B**	65±4	35±2	68±6	/	0.01±1	0.1±7	1.6±5	1.8±5	/
WH	**WH8**	98±3	92	85±2	75±3	0.00±1	0.03±2	0.04±10	0.06±4	0.3±1
	**WH5**	96±2	95.5	95±2	84±7	0.00±0	0.00±2	0.00±2	0.01±2	0.01±3

(A)

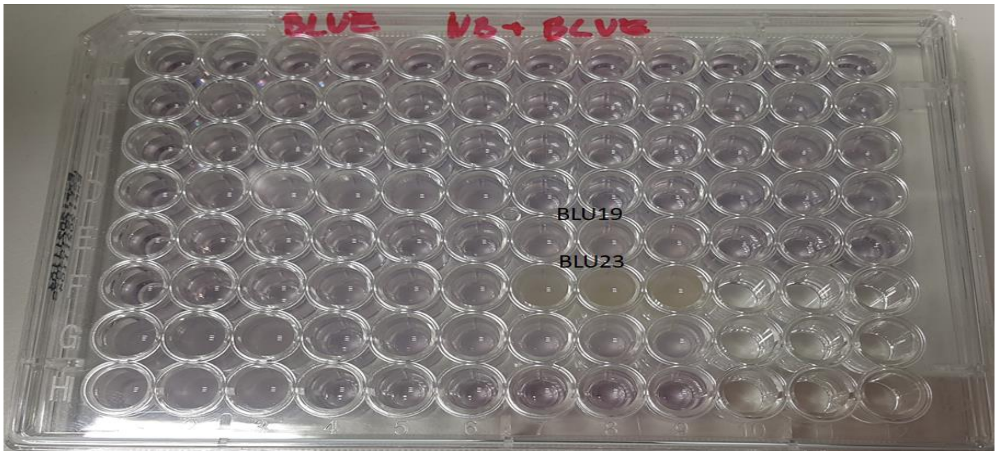

BLUE
NB + BLUE
BLU19
BLU23
G
H

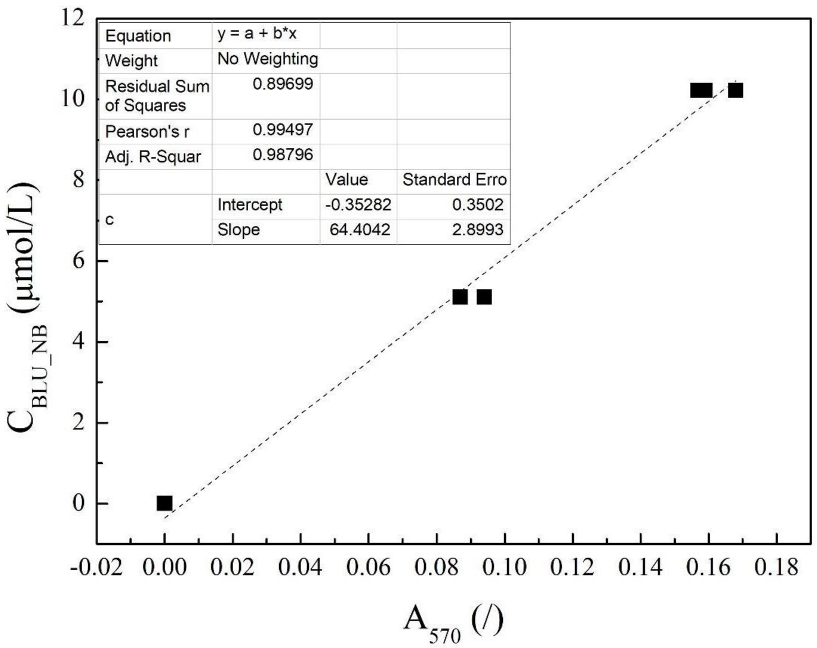

Equation
y = a + b*x
Weight
No Weighting
Residual Sum of Squares
0.89699
Pearson's r
0.99497
Adj. R-Squar
0.98796
Value
Standard Erro
c
Intercept
-0.35282
0.3502
Slope
64.4042
2.8993
C_{BLU_NB} (μmol/L)
A_{570} (/)
0
2
4
6
8
10
12
-0.02
0.00
0.02
0.04
0.06
0.08
0.10
0.12
0.14
0.16
0.18

(B)

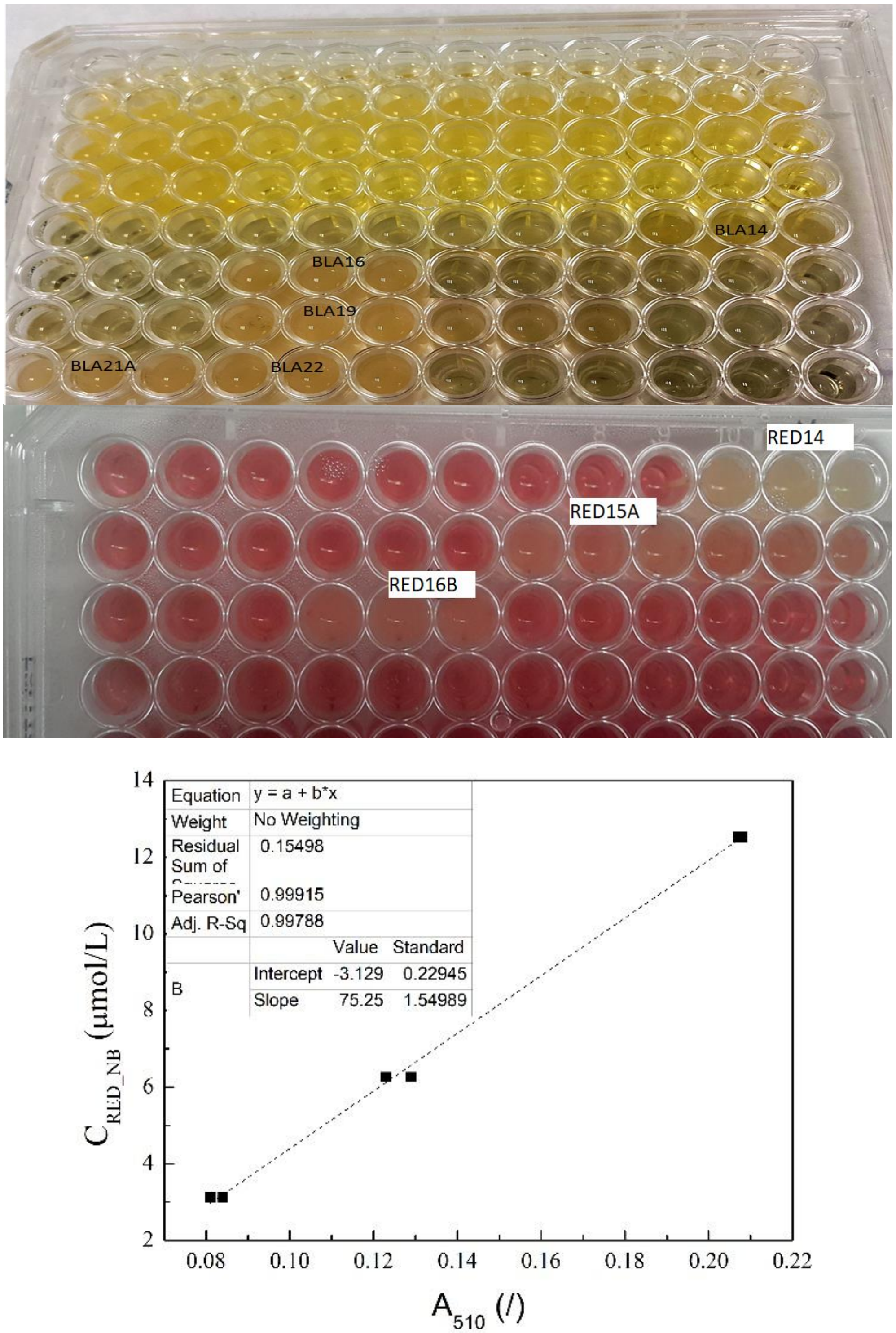
BLA14
BLA16
BLA19
BLA21A
BLA22
RED14
RED15A
RED16B
Equation
y = a + b*x
Weight
No Weighting
Residual Sum of
0.15498
Pearson'
0.99915
Adj. R-Sq
0.99788
Value
Standard
B
Intercept
-3.129
0.22945
Slope
75.25
1.54989
14
12
10
8
6
4
2
0.08
0.10
0.12
0.14
0.16
0.18
0.20
0.22
C_{RED_NB} (μmol/L)
A_{510} (/)

(C)

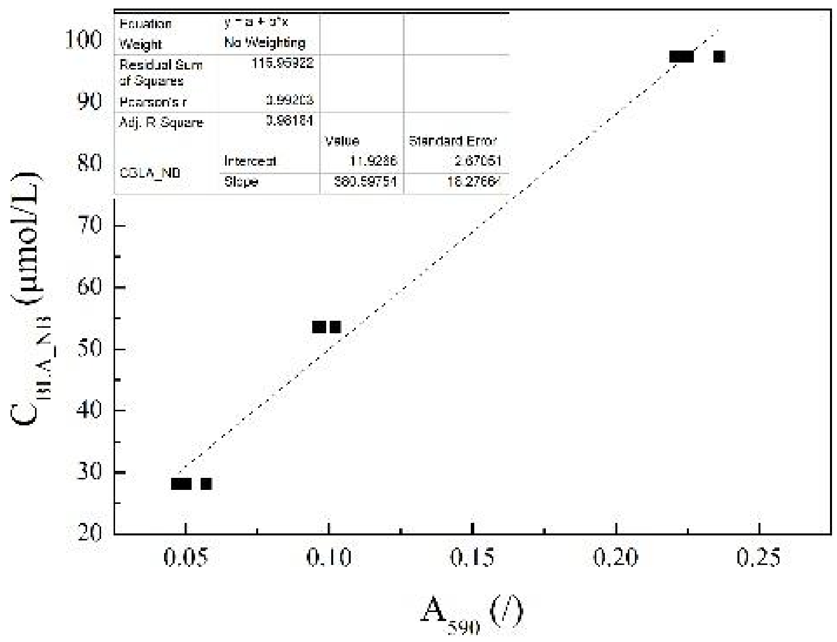

Equation
y = a + b*x
Weight
No Weighting
Residual Sum of Squares
115.95922
Pearson's r
0.99203
Adj. R Square
0.98181
Value
Standard Error
CBLA_NB
Intercept
11.9286
2.67051
Slope
380.59751
16.2766
100
90
80
70
60
50
40
30
20
0.05
0.10
0.15
0.20
0.25
C_BLA_NB (µmol/L)
A_590 (/)

(D)

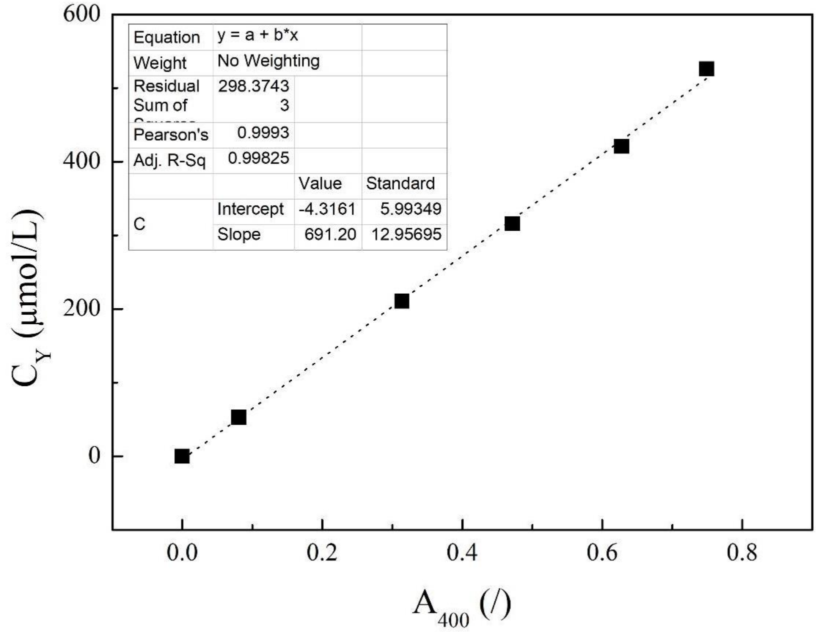

Equation
y = a + b*x
Weight
No Weighting
Residual Sum of
298.3743
3
Pearson's
0.9993
Adj. R-Sq
0.99825
Value
Standard
C
Intercept
-4.3161
5.99349
Slope
691.20
12.95695
600
400
200
0
0.0
0.2
0.4
0.6
0.8
C_Y (µmol/L)
A_400 (/)

(E)

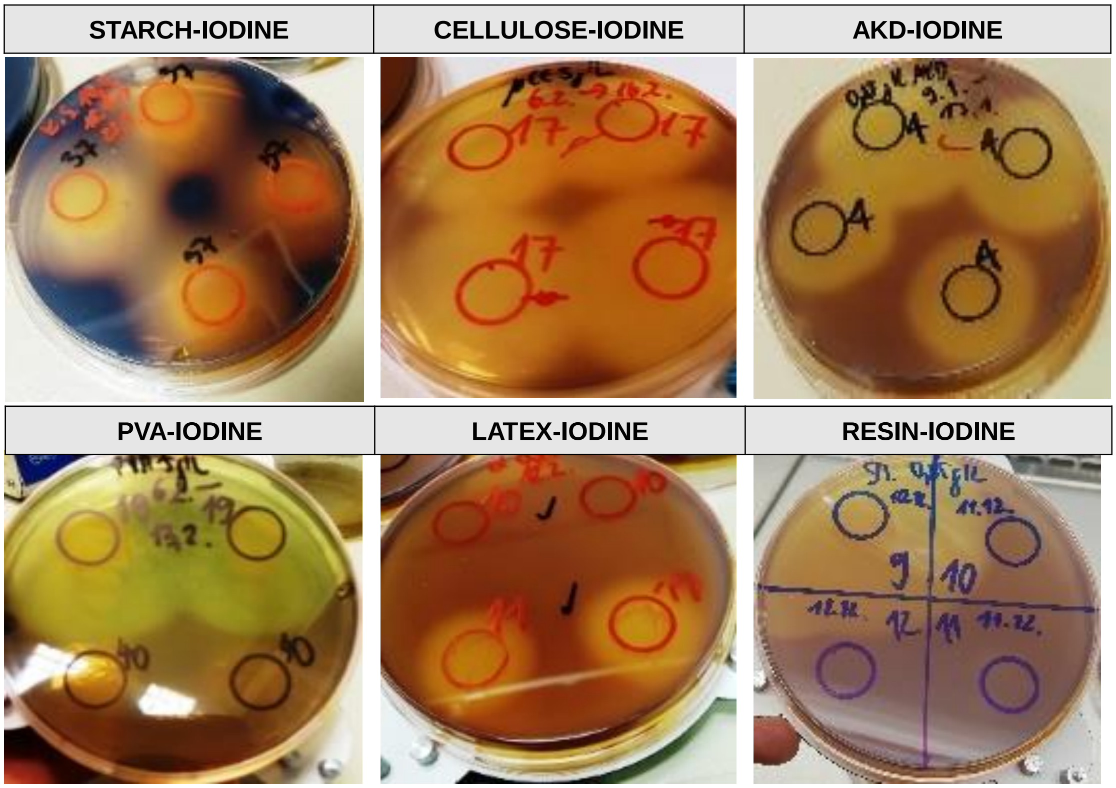

Supplementary Figure A.1. Biodegradation assays: **(A)** above: incubation microtiter plate with 27 BLU isolates in NB with 6 mg/L blue dye, in triplicates, after 18 h at 25°C, below: calibration curve with R^2=0.995; **(B)** above: incubation microtiter plate with 12 RED isolates in NB with 15 mg/L red dye, in triplicates, after 48 h at 25°C, below: calibration curve with R^2=0.999; **(C)** above: microtiter plate rows E-H: 14 BLA isolates in NB with 57 mg/L black dye, in triplicates, after 66 h at 25°C, below: calibration curve for black dye in NB with R^2=0.993; **(D)** calibration curve of yellow dye with R^2=0.999; **(E)** images of M9 agar CST, CE, AKD, PVA, LX and RES after 7-13 days incubation at 25°C without corrections. Above from left to right: Starch-iodine complex formation and distinct discoloring around colonies of CST37, cellulose-iodine complex and distinct discoloring around CE17, AKD-iodine complex and distinct discoloring around AKD4; below from left to right: PVA-iodine complex and distinct discoloring around PVA19 compared to the inactivity of PVA10, LX-iodine complex and distinct discoloration around LX11 compared to the inactivity of LX10 and RES-iodine complex formation and distinct discoloration around RES9 compared to the inactivity of RES10, RES11, RES12.

(A)

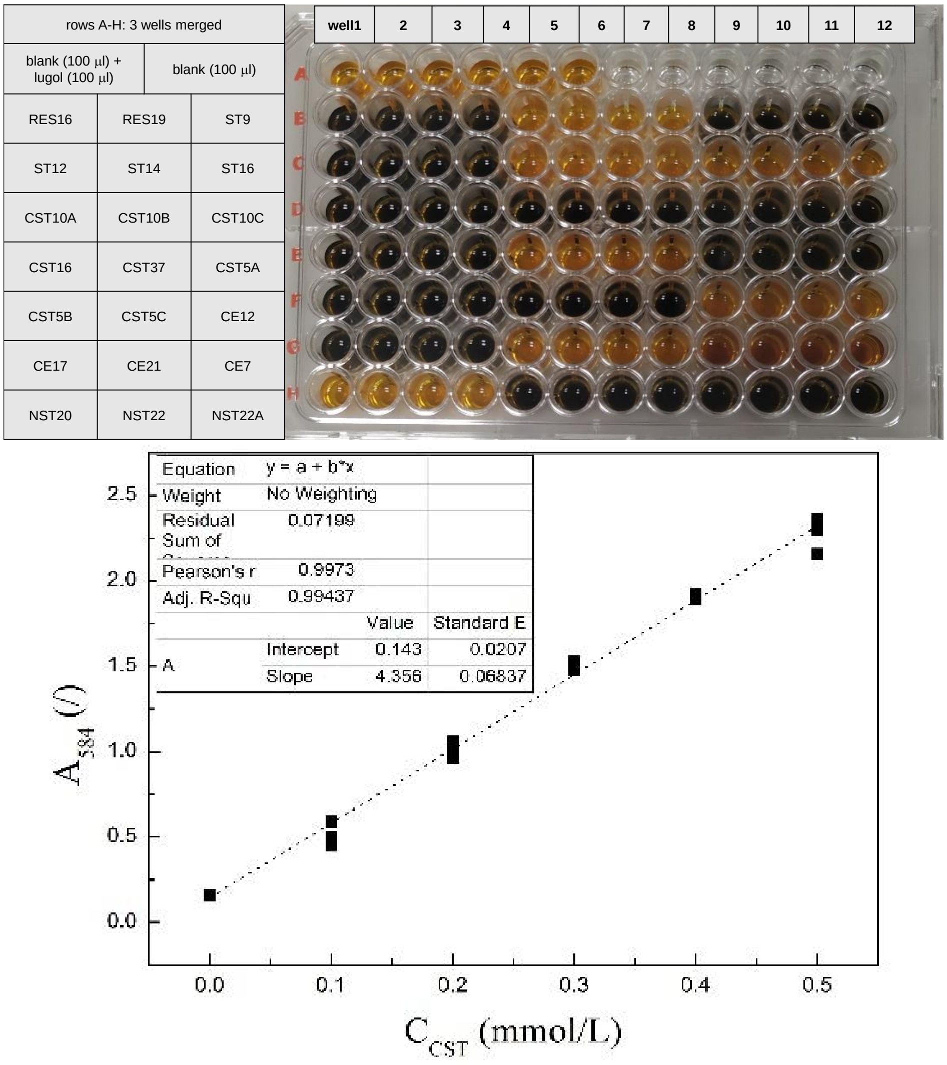
rows A-H: 3 wells merged
blank (100 µl) + lugol (100 µl)
blank (100 µl)
RES16
RES19
ST9
ST12
ST14
ST16
CST10A
CST10B
CST10C
CST16
CST37
CST5A
CST5B
CST5C
CE12
CE17
CE21
CE7
NST20
NST22
NST22A
well1
2
3
4
5
6
7
8
9
10
11
12
Equation
y = a + b*x
Weight
No Weighting
Residual Sum of
0.07199
Pearson's r
0.9973
Adj. R-Squ
0.99437
Value
Standard E
A
Intercept
0.143
0.0207
Slope
4.356
0.06837
A584 (/)
CCST (mmol/L)

(B)

rows A-H		
blank (100 µl) + lugol (100 µl)		blank (100 µl)
RES16	RES19	ST9
ST12	ST14	ST16
CST10A	CST10B	CST10C
CST16	CST37	CST5A
CST5B	CST5C	CE12
CE17	CE21	CE7
NST20	NST22	NST22A

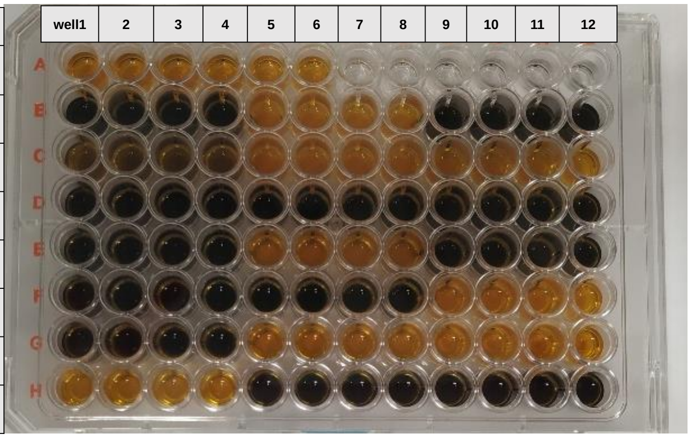

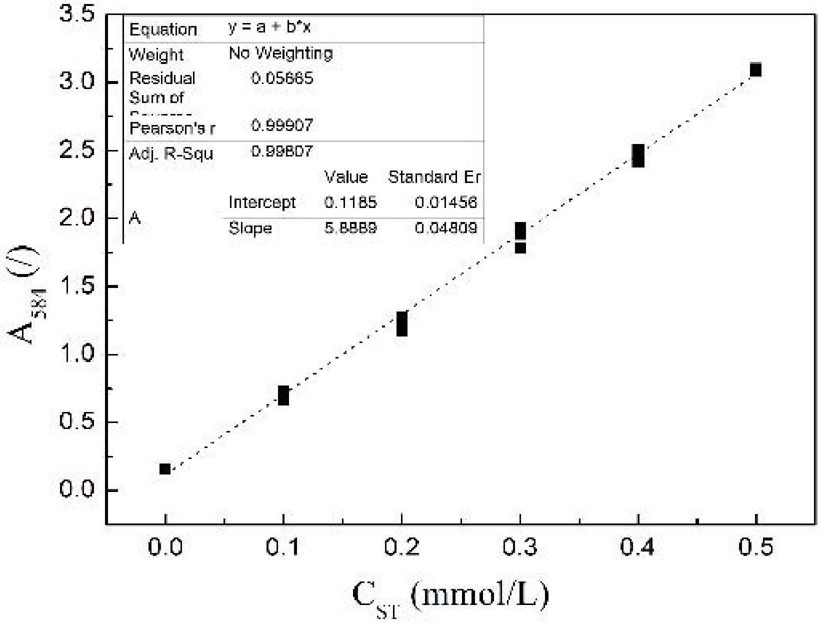

(C)

rows A-H: 3 wells merged		
blank (100 µl) + lugol (100 µl)		control (100 µl) + lugol (100 µl)
RES16	RES19	ST9
ST12	ST14	ST16
CST10A	CST10B	CST10C
CST16	CST37	CST5A
CST5B	CST5C	CST37
CE17	CE21	CE7
NST20	NST22	NST22A

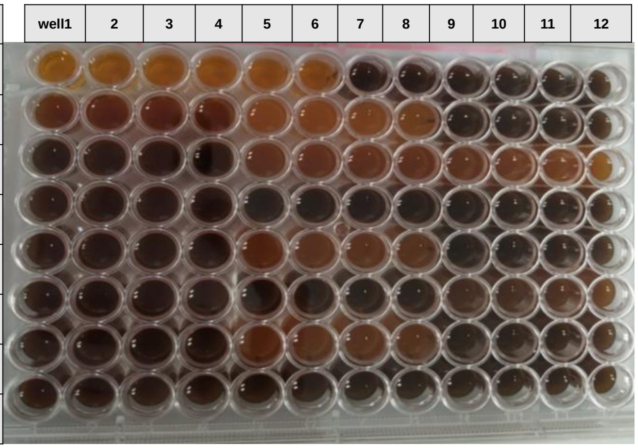

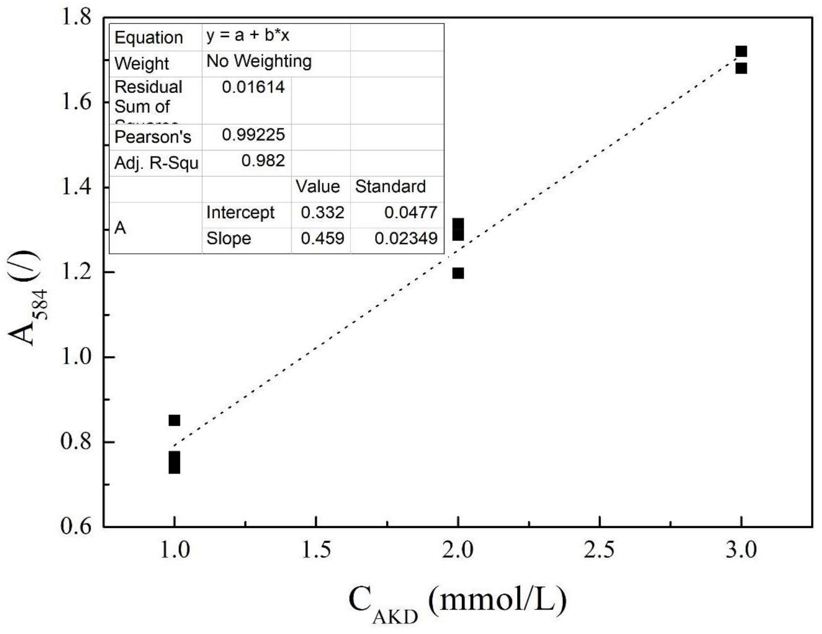
Equation y = a + b*x
Weight No Weighting
Residual Sum of 0.01614
Pearson's 0.99225
Adj. R-Squ 0.982
Value Standard
A Intercept 0.332 0.0477
Slope 0.459 0.02349
A584 (/)
CAKD (mmol/L)
1.8
1.6
1.4
1.2
1.0
0.8
0.6
1.0
1.5
2.0
2.5
3.0

(D)

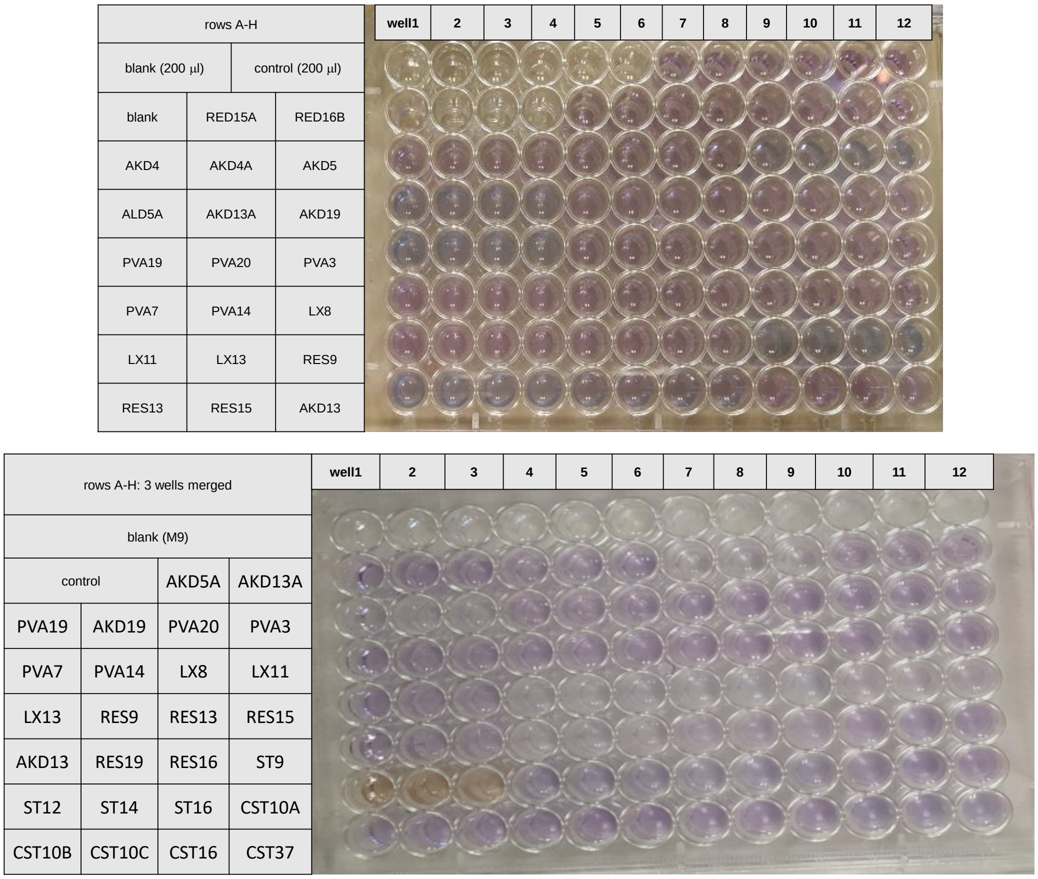
rows A-H
blank (200 µl)
control (200 µl)
blank RED15A RED16B
AKD4 AKD4A AKD5
ALD5A AKD13A AKD19
PVA19 PVA20 PVA3
PVA7 PVA14 LX8
LX11 LX13 RES9
RES13 RES15 AKD13
well1 2 3 4 5 6 7 8 9 10 11 12
rows A-H: 3 wells merged
blank (M9)
control AKD5A AKD13A
PVA19 AKD19 PVA20 PVA3
PVA7 PVA14 LX8 LX11
LX13 RES9 RES13 RES15
AKD13 RES19 RES16 ST9
ST12 ST14 ST16 CST10A
CST10B CST10C CST16 CST37
well1 2 3 4 5 6 7 8 9 10 11 12

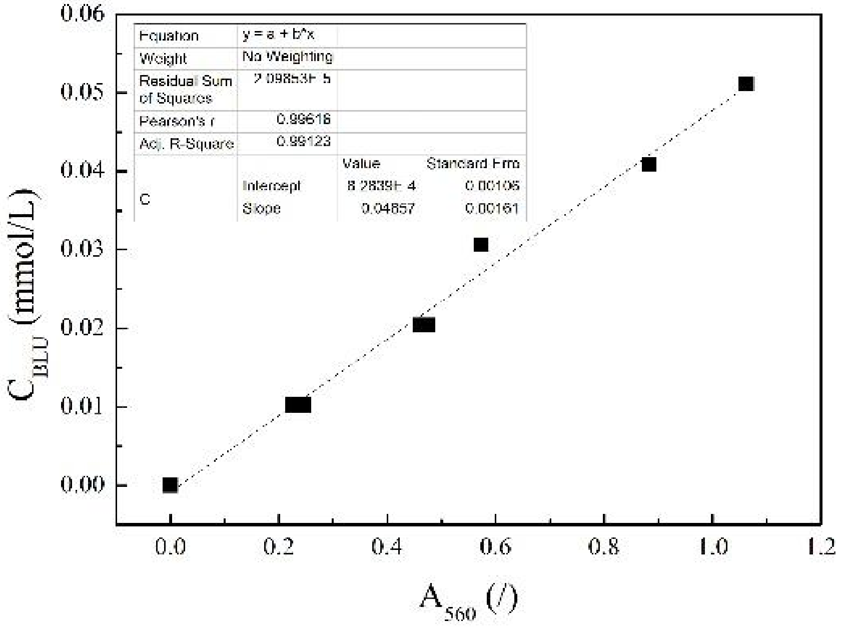
Equation
y = a + b*x
Weight
No Weighting
Residual Sum of Squares
2.09853E-5
Pearson's r
0.99616
Adj. R-Square
0.99123
Value
Standard Error
C
Intercept
8.2839E-4
0.00106
Slope
0.04857
0.00161
0.00
0.01
0.02
0.03
0.04
0.05
0.06
0.0
0.2
0.4
0.6
0.8
1.0
1.2
C_{BLU} (mmol/L)
A_{560} (/)

(E)

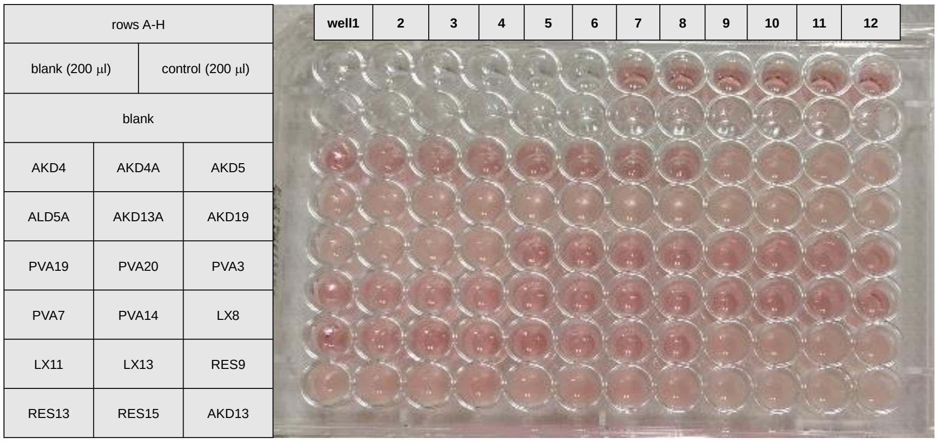
rows A-H
blank (200 µl)
control (200 µl)
blank
AKD4
AKD4A
AKD5
ALD5A
AKD13A
AKD19
PVA19
PVA20
PVA3
PVA7
PVA14
LX8
LX11
LX13
RES9
RES13
RES15
AKD13
well1
2
3
4
5
6
7
8
9
10
11
12

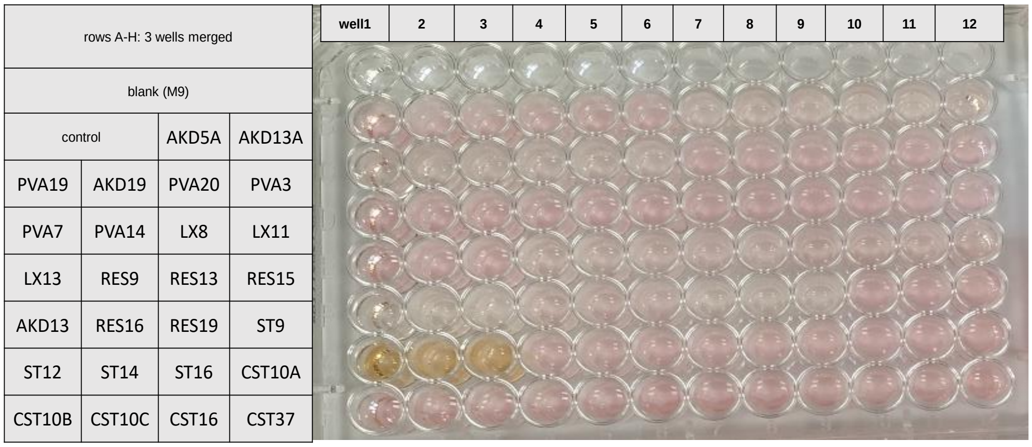
rows A-H: 3 wells merged
blank (M9)
control
AKD5A
AKD13A
PVA19
AKD19
PVA20
PVA3
PVA7
PVA14
LX8
LX11
LX13
RES9
RES13
RES15
AKD13
RES16
RES19
ST9
ST12
ST14
ST16
CST10A
CST10B
CST10C
CST16
CST37
well1
2
3
4
5
6
7
8
9
10
11
12

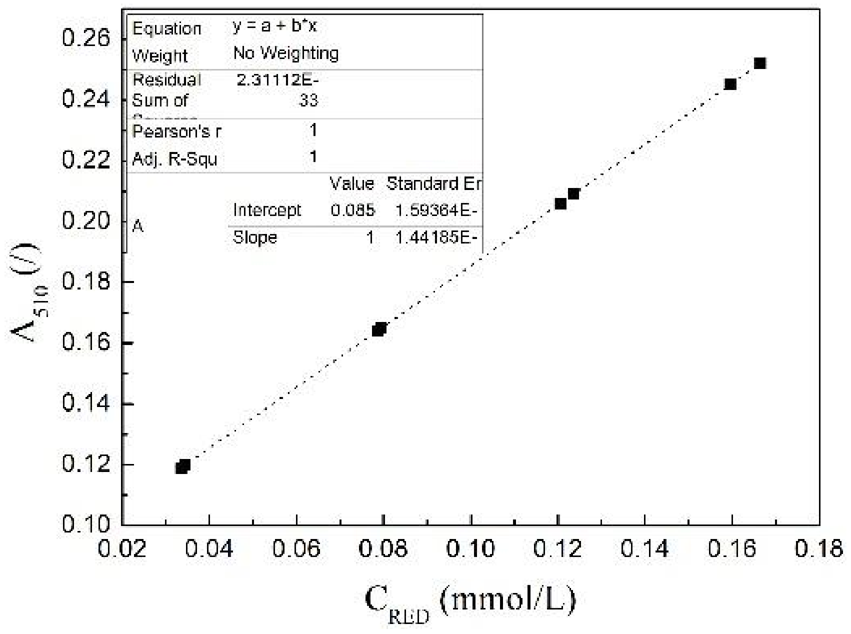
Equation y = a + b*x
Weight No Weighting
Residual Sum of 2.31112E-33
Pearson's r 1
Adj. R-Squ 1
Value Standard Er
A Intercept 0.085 1.59364E-
Slope 1 1.44185E-
0.26
0.24
0.22
0.20
0.18
0.16
0.14
0.12
0.10
0.02 0.04 0.06 0.08 0.10 0.12 0.14 0.16 0.18
A_{510} (/)
C_{RED} (mmol/L)

(F)

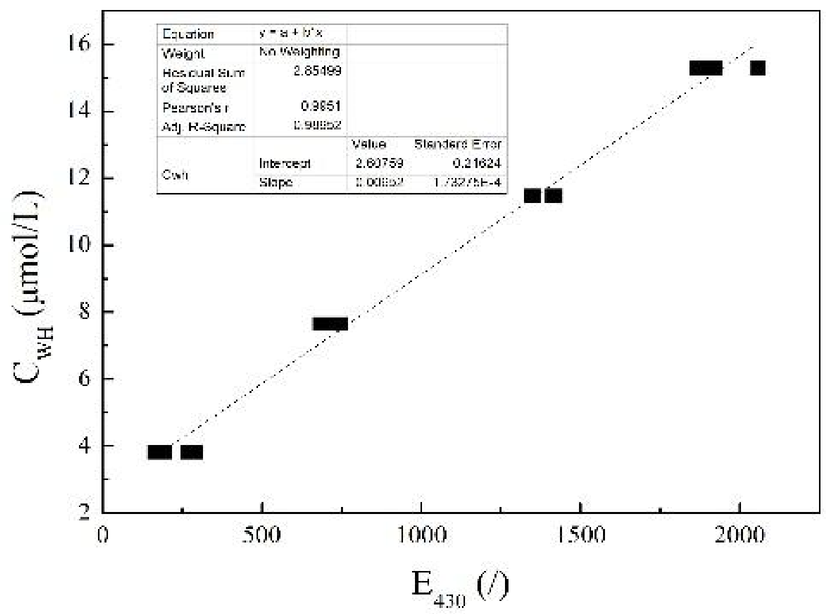
16
14
12
10
8
6
4
2
0 500 1000 1500 2000
C_{WH} (μmol/L)
E_{430} (/)

(G)

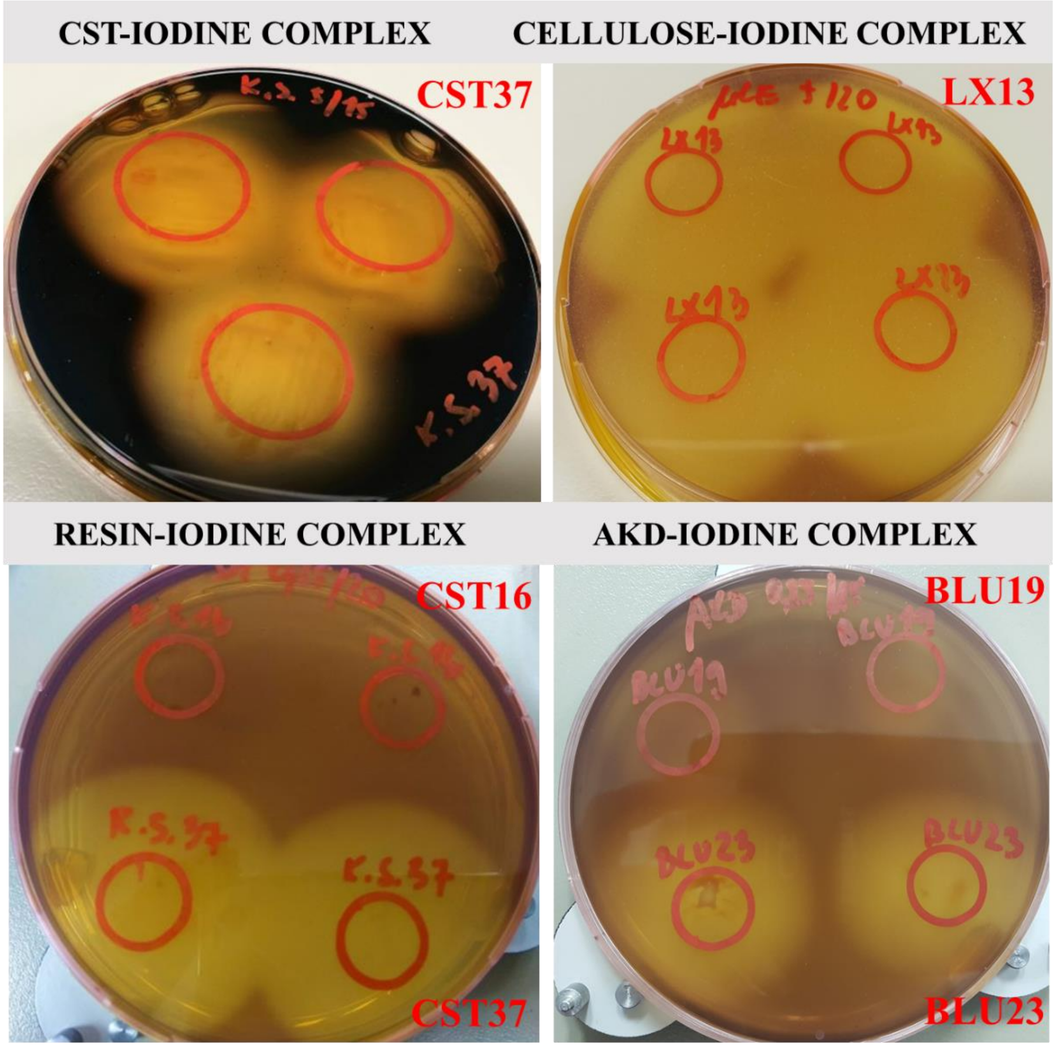

Supplementary Figure A.2. Tests for reservoir of carbon source usage. **(A)** M9 0.5 g/L CST: above: microtiter plate after addition of Lugol, below: calibration curve (R^2=0.997); **(B)** M9 0.5 g/L ST: above: microtiter plate after addition of Lugol, below: calibration curve (R^2=0.999); **(C)** M9 4 g/L AKD: above: microtiter plate after addition of Lugol, below: calibration curve (R^2=0.999); **(D)** BLU in $M9_{Glc}$: above: microtiter plate after 4 days at 25°C, middle: supernatants, below: calibration curve (R^2=0.996); **(E)** RED in $M9_{Glc}$: above: microtiter plate after 4 days at 25°C, middle: supernatants, below: calibration curve (R^2=0.999); **(F)** WH in $M9_{Glc}$ calibration curve (R^2=0.996); **(G)** above from left to right: Cationic starch-iodine complex formation after addition of Lugol and distinct discoloration around CST37. Cellulose-iodine complex formation and discoloration due to activities of LX13. Below from left to right: Resin-iodine formation and distinctive discoloration around colonies of CST37 in comparison to inactivity of CST16. AKD-iodine complex formation and discoloration around colonies of BLU23 in comparison to only slight activity of BLU19.

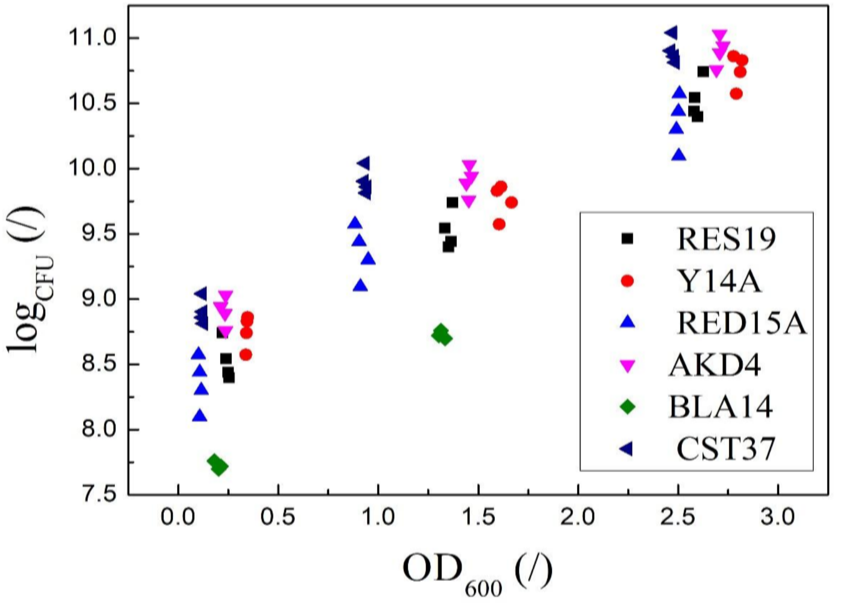

Supplementary Figure A.3. Calibration of the growth of the 6 selected bacteria; log(CFU) vs. OD_{600}.

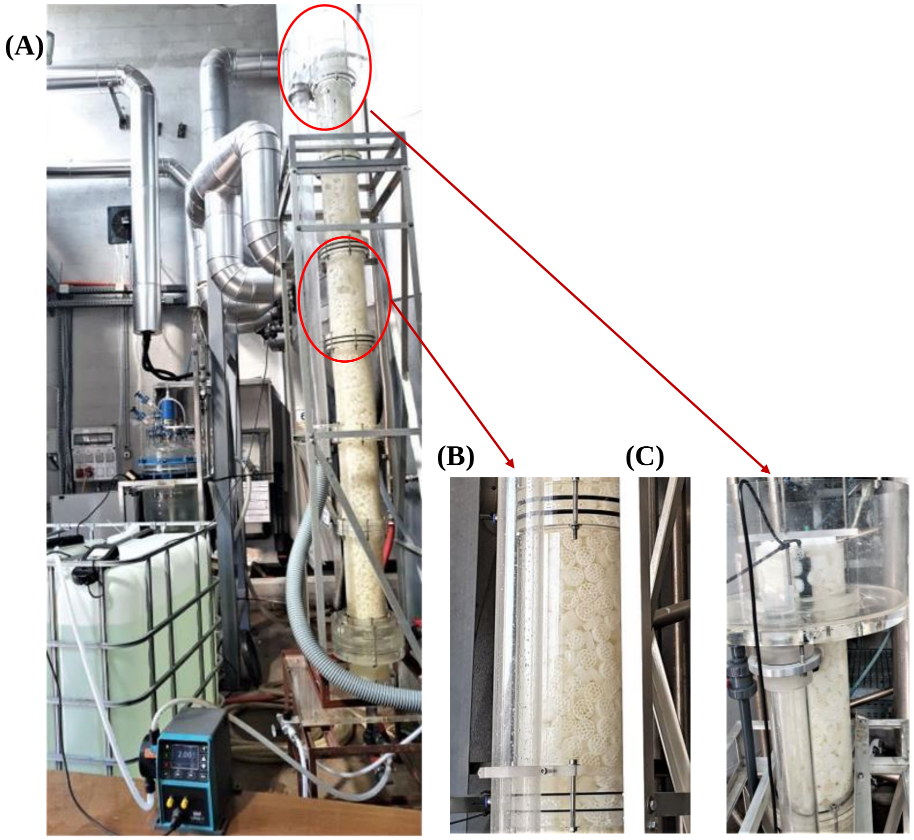

(D)

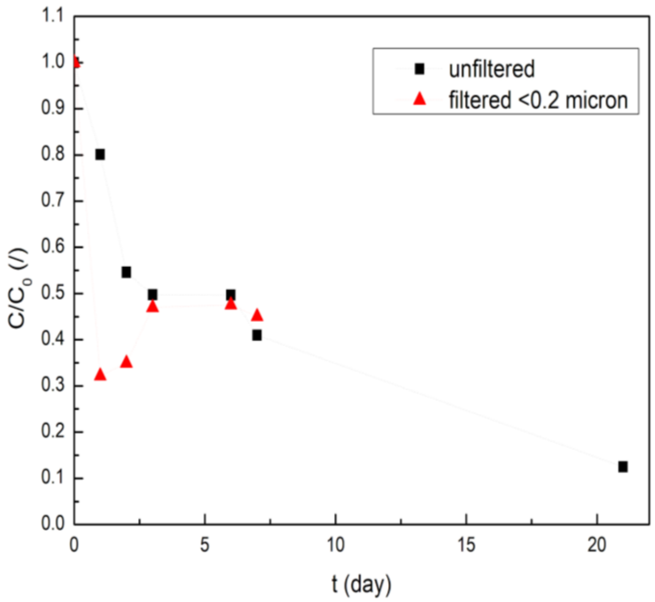

(E)

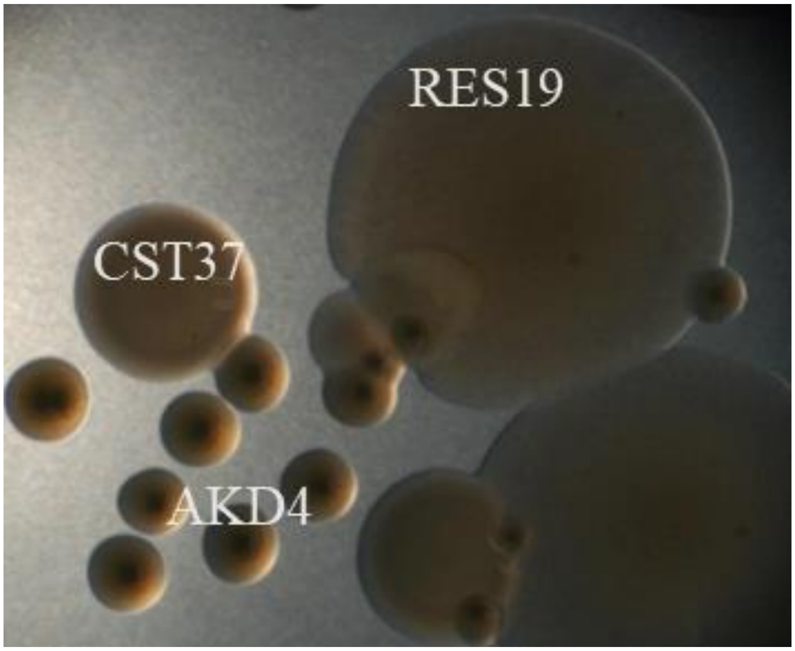

Supplementary Figure A.4. Pilot test (for proof of concept only): **(A)** Photograph of the set-up: a 33 L column with parallel flow of whitewater and air entering at the bottom of the column; **(B)** part of the column filled with plastic carriers "Kaldens"; **(C)** upper part of the column with inserted oxygen electrode; **(D)** C/C_0 from COD measurements of influent and effluent whitewater as a function of time; whitewater samples unfiltered and filtered through 0.2 μm membrane, **(E)** A representative image of

the colonies obtained with the microscope Motic SMZ-168- BL in a parfocal 6.7:1 zoom. The CFU count was as follows: 3×RES19, 2×CST37 and 13×AKD4.

(A)

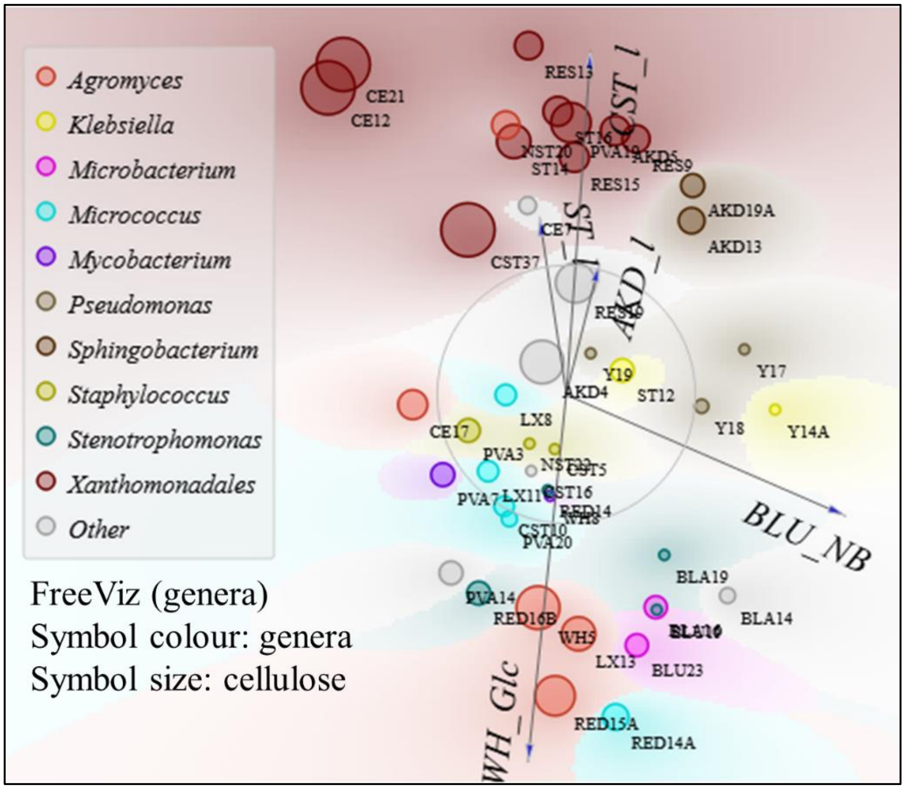

(B)

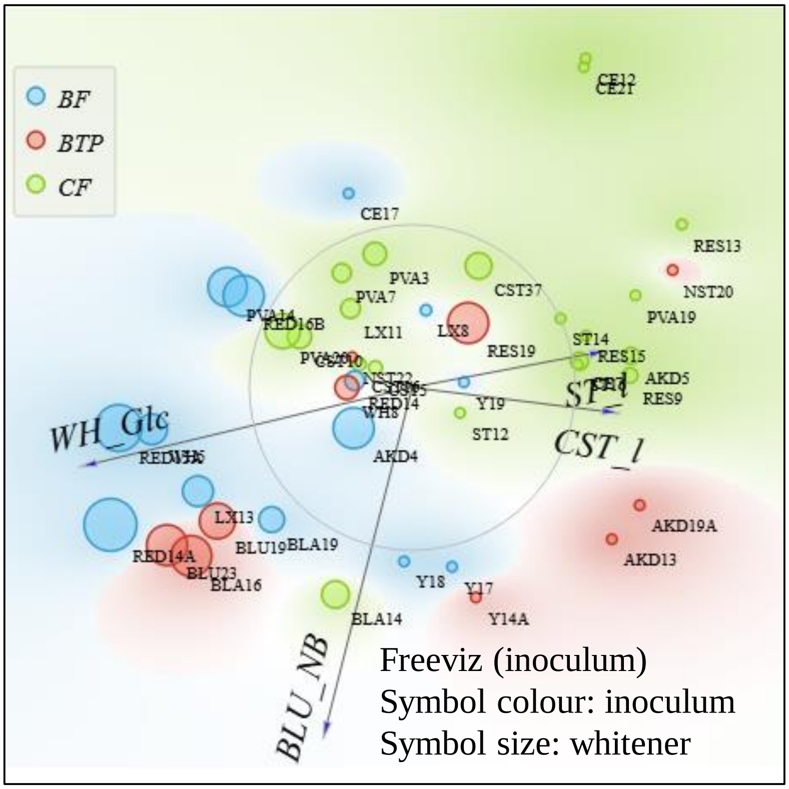
BF
BTP
CF
CE17
RES13
NST20
PVA19
PVA3
PVA7
CST37
LX11
LX8
RES19
ST14
RES15
AKD5
RES9
Y19
ST12
AKD4
WH_Glc
CST_1
AKD19A
AKD13
LX13
BLU19
BLA19
RED14A
BLA16
Y18
Y17
Y14A
BLA14
BLU_NB
Freeviz (inoculum)
Symbol colour: inoculum
Symbol size: whitener

(C)

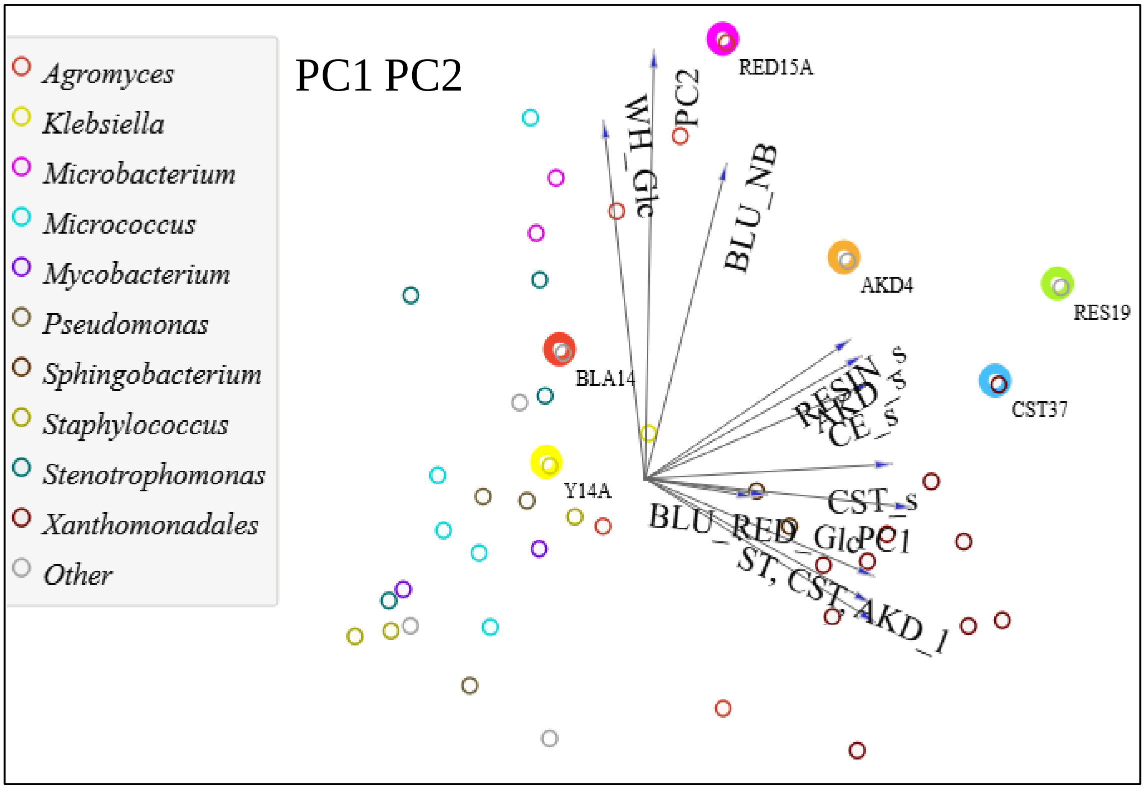
Agromyces
Klebsiella
Microbacterium
Micrococcus
Mycobacterium
Pseudomonas
Sphingobacterium
Staphylococcus
Stenotrophomonas
Xanthomonadales
Other
PC1 PC2
RED15A
PC2
WH_Glc
BLU_NB
AKD4
RES19
BLA14
CST37
Y14A
CST_s

(D)

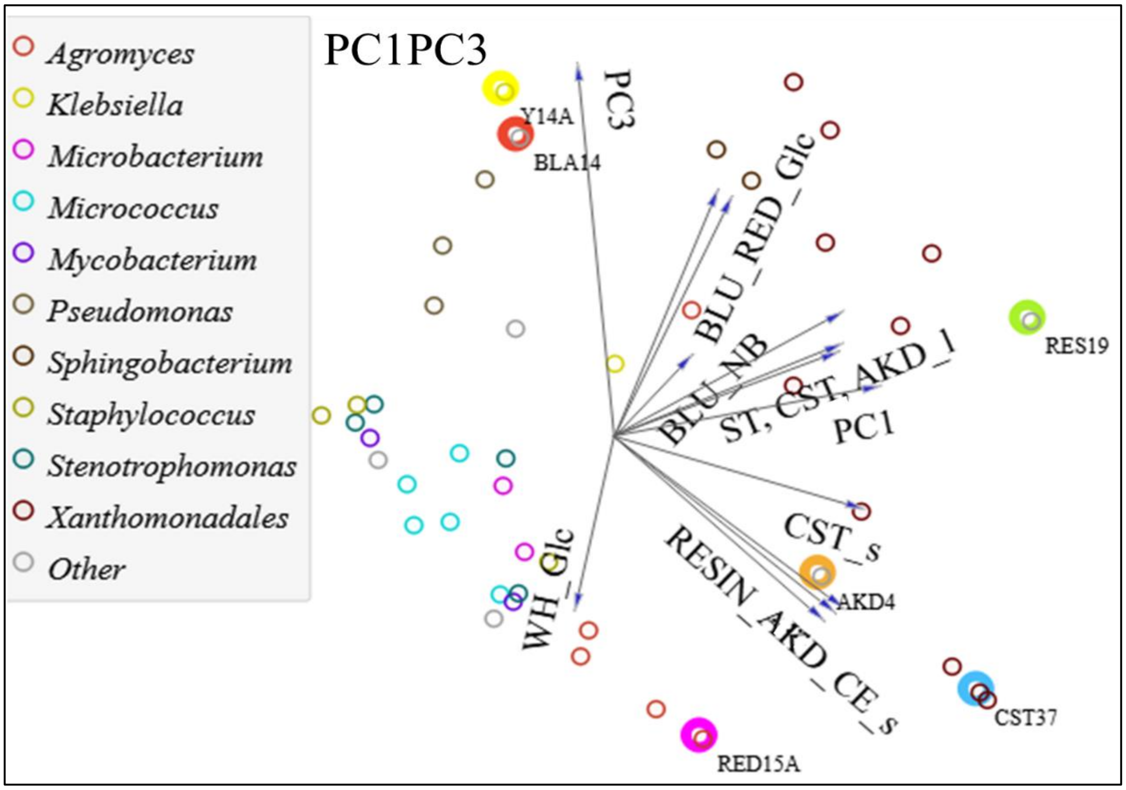

Supplementary Figure A.5. FreeViz and PCA of tests for the repertoire of carbon sources of 44 isolates in liquid and solid media. FreeViz projection with (**A)** clusters of genera and symbol size for cellulose and (**B)** clusters of inoculum and symbol size for whitener degradation, PCA with clusters for genera and marked 6 selected strains, RED15A (circled in pink), BLA14 (blue), Y14A (yellow), RES19 (green), AKD4 (brown) and CST37 (red): (**C)** PC1PC2 with 67% and (**D)** PC1PC3 with 65% explained variances.

(A)

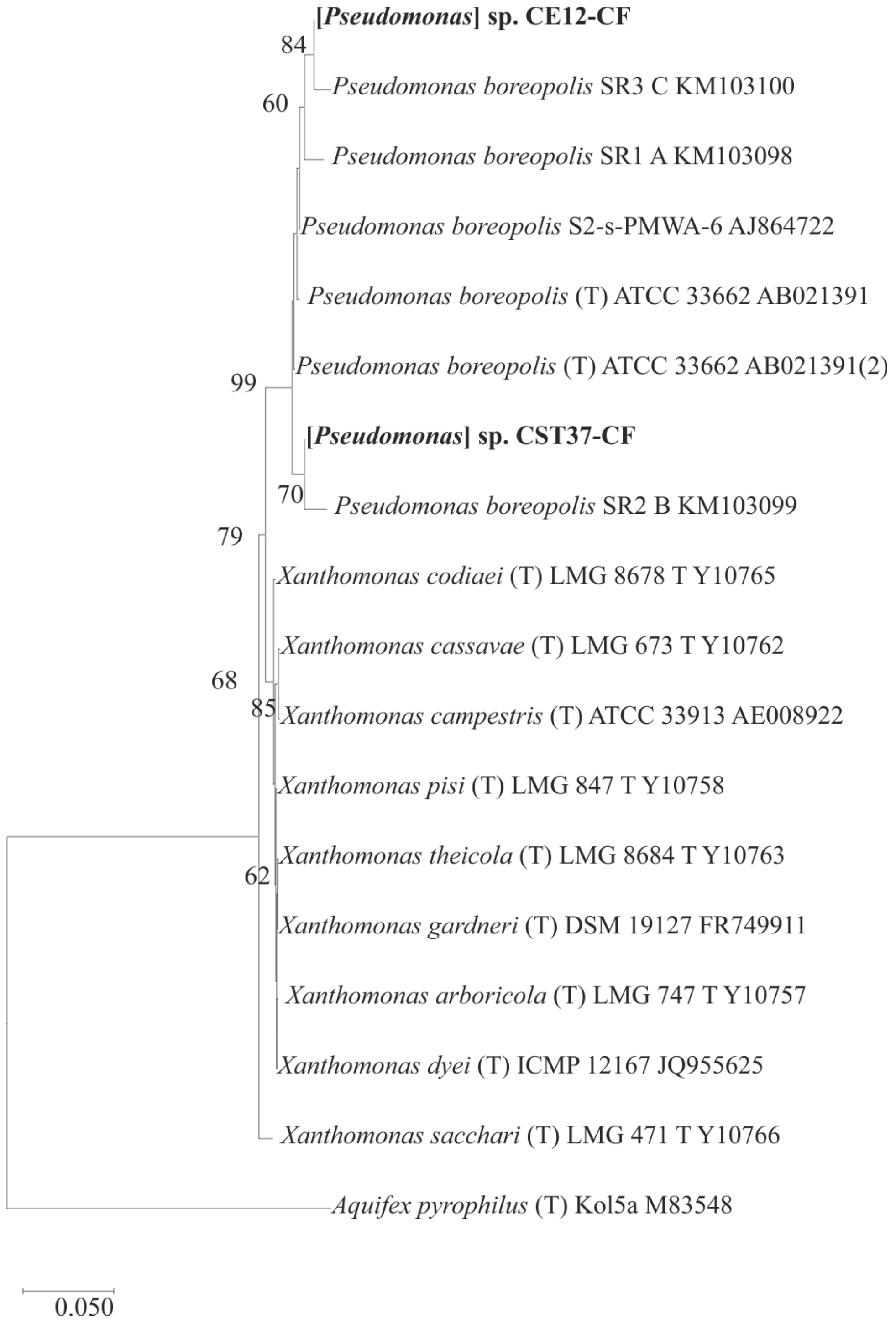
[Pseudomonas] sp. CE12-CF
Pseudomonas boreopolis SR3 C KM103100
Pseudomonas boreopolis SR1 A KM103098
Pseudomonas boreopolis S2-s-PMWA-6 AJ864722
Pseudomonas boreopolis (T) ATCC 33662 AB021391
Pseudomonas boreopolis (T) ATCC 33662 AB021391(2)
[Pseudomonas] sp. CST37-CF
Pseudomonas boreopolis SR2 B KM103099
Xanthomonas codiaei (T) LMG 8678 T Y10765
Xanthomonas cassavae (T) LMG 673 T Y10762
Xanthomonas campestris (T) ATCC 33913 AE008922
Xanthomonas pisi (T) LMG 847 T Y10758
Xanthomonas theicola (T) LMG 8684 T Y10763
Xanthomonas gardneri (T) DSM 19127 FR749911
Xanthomonas arboricola (T) LMG 747 T Y10757
Xanthomonas dyei (T) ICMP 12167 JQ955625
Xanthomonas sacchari (T) LMG 471 T Y10766
Aquifex pyrophilus (T) Kol5a M83548
84
60
99
70
79
68
85
62
0.050

(B)

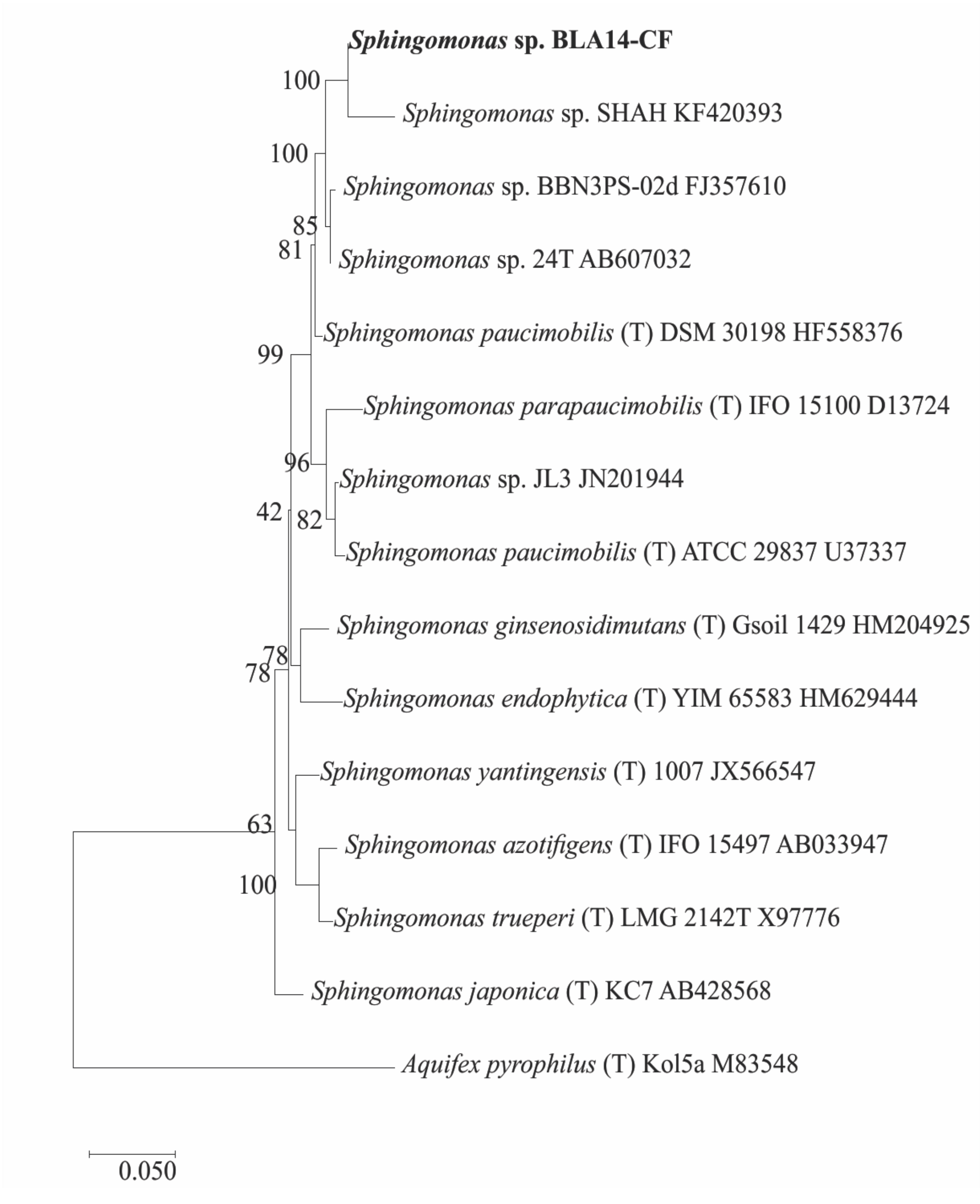
Sphingomonas sp. BLA14-CF
Sphingomonas sp. SHAH KF420393
Sphingomonas sp. BBN3PS-02d FJ357610
Sphingomonas sp. 24T AB607032
Sphingomonas paucimobilis (T) DSM 30198 HF558376
Sphingomonas parapaucimobilis (T) IFO 15100 D13724
Sphingomonas sp. JL3 JN201944
Sphingomonas paucimobilis (T) ATCC 29837 U37337
Sphingomonas ginsenosidimutans (T) Gsoil 1429 HM204925
Sphingomonas endophytica (T) YIM 65583 HM629444
Sphingomonas yantingensis (T) 1007 JX566547
Sphingomonas azotifigens (T) IFO 15497 AB033947
Sphingomonas trueperi (T) LMG 2142T X97776
Sphingomonas japonica (T) KC7 AB428568
Aquifex pyrophilus (T) Kol5a M83548
100
100
85
81
99
96
42
82
78
78
63
100
0.050

(C)

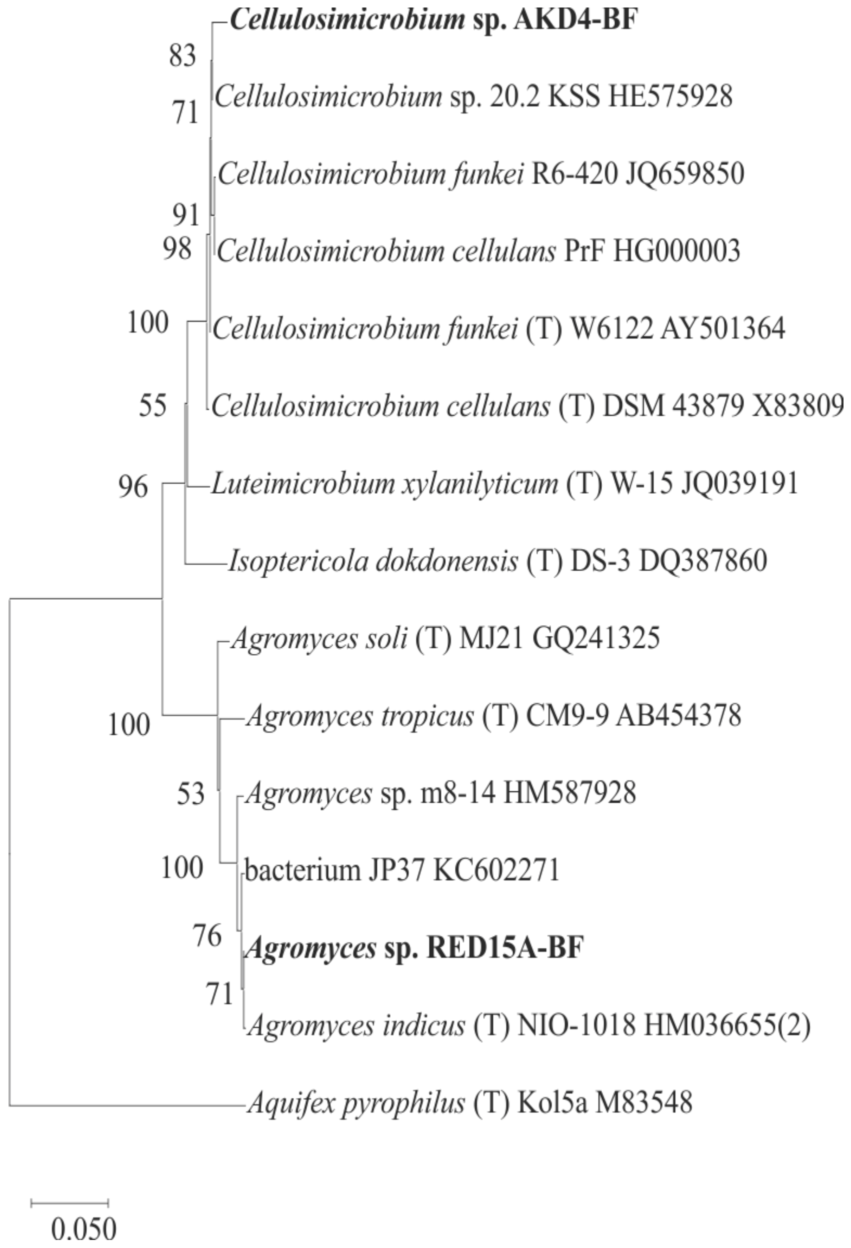
Cellulosimicrobium sp. AKD4-BF
83
Cellulosimicrobium sp. 20.2 KSS HE575928
71
Cellulosimicrobium funkei R6-420 JQ659850
91
98
Cellulosimicrobium cellulans PrF HG000003
100
Cellulosimicrobium funkei (T) W6122 AY501364
55
Cellulosimicrobium cellulans (T) DSM 43879 X83809
96
Luteimicrobium xylanilyticum (T) W-15 JQ039191
Isoptericola dokdonensis (T) DS-3 DQ387860
Agromyces soli (T) MJ21 GQ241325
100
Agromyces tropicus (T) CM9-9 AB454378
53
Agromyces sp. m8-14 HM587928
100
bacterium JP37 KC602271
76
Agromyces sp. RED15A-BF
71
Agromyces indicus (T) NIO-1018 HM036655(2)
Aquifex pyrophilus (T) Kol5a M83548
0.050

(D)

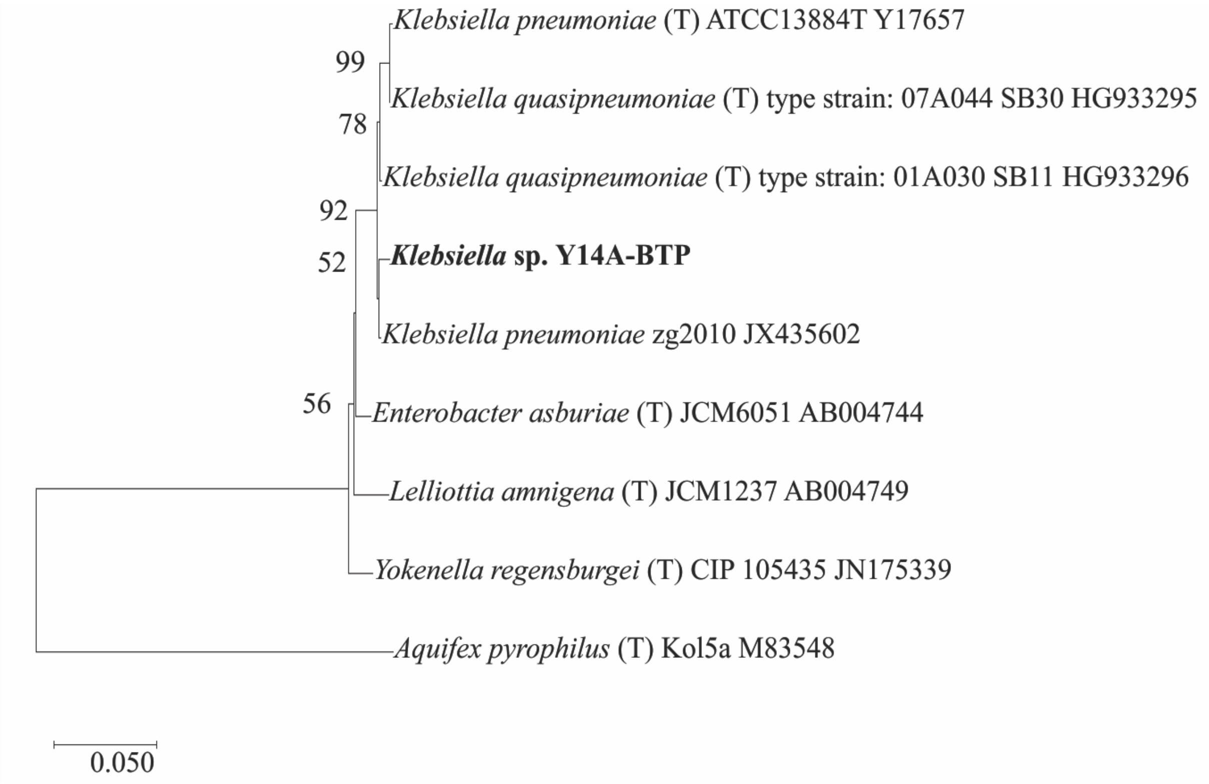
Klebsiella pneumoniae (T) ATCC13884T Y17657
99
Klebsiella quasipneumoniae (T) type strain: 07A044 SB30 HG933295
78
Klebsiella quasipneumoniae (T) type strain: 01A030 SB11 HG933296
92
Klebsiella sp. Y14A-BTP
52
Klebsiella pneumoniae zg2010 JX435602
56
Enterobacter asburiae (T) JCM6051 AB004744
Lelliottia amnigena (T) JCM1237 AB004749
Yokenella regensburgei (T) CIP 105435 JN175339
Aquifex pyrophilus (T) Kol5a M83548
0.050

(E)

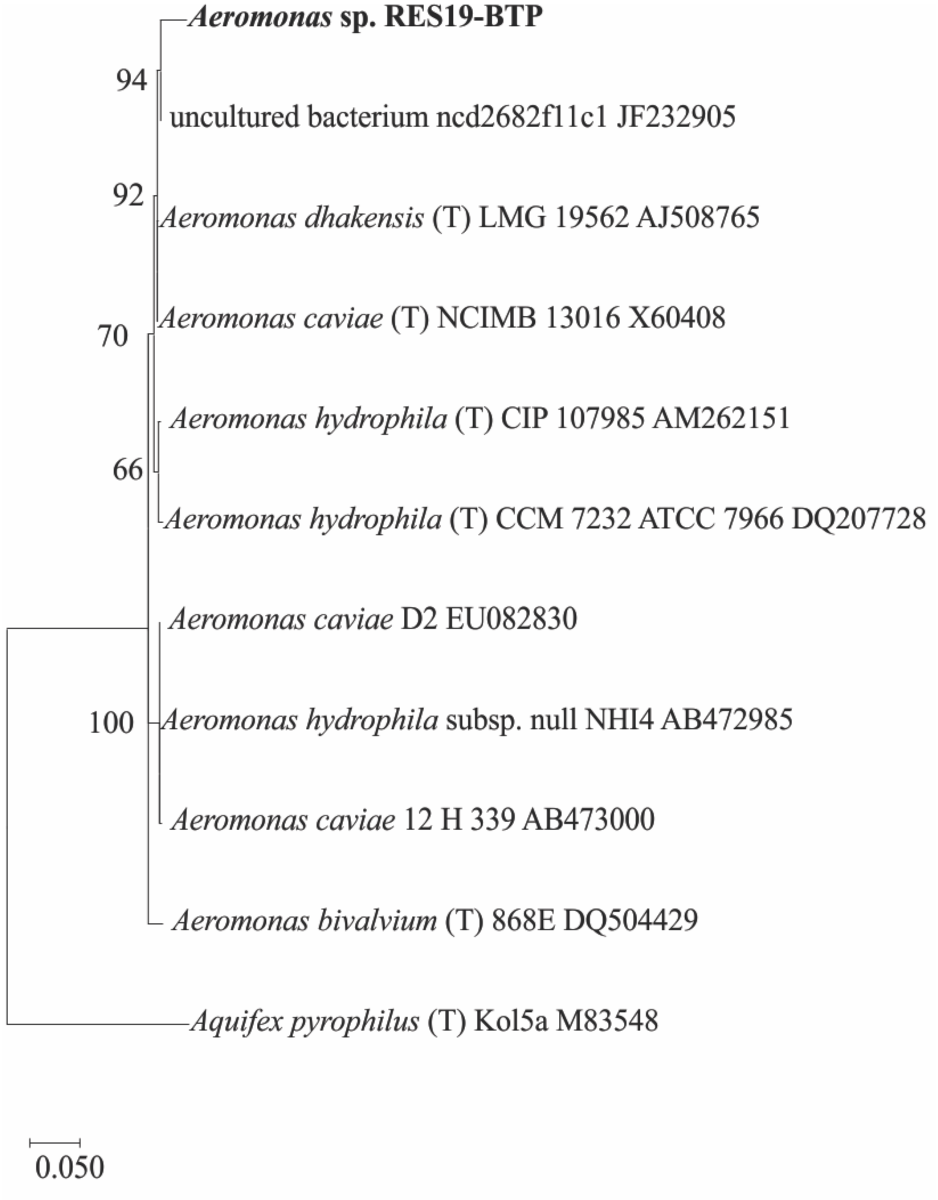

Supplementary Figure A.6. Detailed phylogenetic trees displaying the phylogenetic position of six selected isolates with their closest neighbors are presented. *Aquifex pyrophilus* (T) was used as an outlier. Numbers indicate the percentages of bootstrap support, derived from 1000 re-samplings. The evolutionary history was derived using the Neighbor-Joining method (Saitou and Nei, 1987). **(A)** Xanthomonadales bacterium strain CST37, **(B)** *Sphingomonas* sp. strain BLA14-CF, **(C)** *Cellulosimicrobium* sp. strain AKD4-BF together with *Agromyces* sp. strain RED15A, **(D)** *Klebsiella* sp. strain Y14A-BTP and **(E)** *Aeromonas* sp. RES19-BTP.
